# Supplementary material for: An integrated view of the structure and function of the human 4D nucleome
Source: Nature. 2025 Dec 17;649(8097):759–76. doi: 10.1038/s41586-025-09890-3 (PMC12804090; doi:10.1038/s41586-025-09890-3)
Supplement: Supplementary file 1 — Supplementary Figures 1-13, Supplementary Table 1 and Supplementary Note 1 [file 41586_2025_9890_MOESM1_ESM.pdf]

nature portfolio

https://doi.org/10.1038/s41586-025-09890-3

Supplementary information

An integrated view of the structure and function of the human 4D nucleome

In the format provided by the authors and unedited

Supplementary Table I: List of companion papers

PMID	Reference
29522506	Barton, C., Morganella, S., Odegard-Fougner, O., Alexander, S., Ries, J., Fitzgerald, T., Ellenberg, J., and Birney, E. (2018). ChromoTrace: Computational reconstruction of 3D chromosome configurations for super-resolution microscopy. PLoS Comput Biol 14, e1006002. 10.1371/journal.pcbi.1006002.
32358536	Trzaskoma, P., Ruszczycki, B., Lee, B., Pels, K.K., Krawczyk, K., Bokota, G., Szczepankiewicz, A.A., Aaron, J., Walczak, A., Sliwinska, M.A., et al. (2020). Ultrastructural visualization of 3D chromatin folding using volume electron microscopy and DNA in situ hybridization. Nat Commun 11, 2120. 10.1038/s41467-020-15987-2.
33446254	Wang, Y., Zhang, Y., Zhang, R., van Schaik, T., Zhang, L., Sasaki, T., Peric-Hupkes, D., Chen, Y., Gilbert, D.M., van Steensel, B., et al. (2021). SPIN reveals genome-wide landscape of nuclear compartmentalization. Genome Biol 22, 36. 10.1186/s13059-020-02253-3.
33963348	Fiorillo, L., Musella, F., Conte, M., Kempfer, R., Chiariello, A.M., Bianco, S., Kukalev, A., Irastorza-Azcarate, I., Esposito, A., Abraham, A., et al. (2021). Comparison of the Hi-C, GAM and SPRITE methods using polymer models of chromatin. Nat Methods 18, 482-490. 10.1038/s41592-021-01135-1.
34106828	Strom, A.R., Biggs, R.J., Banigan, E.J., Wang, X., Chiu, K., Herman, C., Collado, J., Yue, F., Ritland Politz, J.C., Tait, L.J., et al. (2021). HP1alpha is a chromatin crosslinker that controls nuclear and mitotic chromosome mechanics. Elife 10. 10.7554/eLife.63972.
34446921	Yu, M., Abnousi, A., Zhang, Y., Li, G., Lee, L., Chen, Z., Fang, R., Lagler, T.M., Yang, Y., Wen, J., et al. (2021). SnapHiC: a computational pipeline to identify chromatin loops from single-cell Hi-C data. Nat Methods 18, 1056-1059. 10.1038/s41592-021-01231-2.
34480151	Akgol Oksuz, B., Yang, L., Abraham, S., Venev, S.V., Krietenstein, N., Parsi, K.M., Ozadam, H., Oomen, M.E., Nand, A., Mao, H., et al. (2021). Systematic evaluation of chromosome conformation capture assays. Nat Methods 18, 1046-1055. 10.1038/s41592-021-01248-7.
34635838	Zhang, R., Zhou, T., and Ma, J. (2022). Multiscale and integrative single-cell Hi-C analysis with Higashi. Nat Biotechnol 40, 254-261. 10.1038/s41587-021-01034-y.
34663924	Janssens, D.H., Meers, M.P., Wu, S.J., Babaeva, E., Meshinchi, S., Sarthy, J.F., Ahmad, K., and Henikoff, S. (2021). Automated CUT&Tag profiling of chromatin heterogeneity in mixed-lineage leukemia. Nat Genet 53, 1586-1596. 10.1038/s41588-021-00941-9.
34789882	Winick-Ng, W., Kukalev, A., Harabula, I., Zea-Redondo, L., Szabo, D., Meijer, M., Serebreni, L., Zhang, Y., Bianco, S., Chiariello, A.M., et al. (2021). Cell-type specialization is encoded by specific chromatin topologies. Nature 599, 684-691. 10.1038/s41586-021-04081-2.
34862501	Hammer, M., Huisman, M., Rigano, A., Boehm, U., Chambers, J.J., Gaudreault, N., North, A.J., Pimentel, J.A., Sudar, D., Bajcsy, P., et al. (2021). Towards community-driven metadata standards for light microscopy: tiered specifications extending the OME model. Nat Methods 18, 1427-1440. 10.1038/s41592-021-01327-9.
34862503	Rigano, A., Ehmsen, S., Ozturk, S.U., Ryan, J., Balashov, A., Hammer, M., Kirli, K., Boehm, U., Brown, C.M., Bellve, K., et al. (2021). Micro-Meta App: an interactive tool for collecting microscopy metadata based on community specifications. Nat Methods 18, 1489-1495. 10.1038/s41592-021-01315-z.
35412637	Li, D., Purushotham, D., Harrison, J.K., Hsu, S., Zhuo, X., Fan, C., Liu, S., Xu, V., Chen, S., Xu, J., et al. (2022). WashU Epigenome Browser update 2022. Nucleic Acids Res 50, W774-W781. 10.1093/nar/gkac238.
35440119	Feng, F., Yao, Y., Wang, X.Q.D., Zhang, X., and Liu, J. (2022). Connecting high-resolution 3D chromatin organization with epigenomics. Nat Commun 13, 2054. 10.1038/s41467-022-29695-6.
35501320	Reiff, S.B., Schroeder, A.J., Kirli, K., Cosolo, A., Bakker, C., Mercado, L., Lee, S., Veit, A.D., Balashov, A.K., Vitzthum, C., et al. (2022). The 4D Nucleome Data Portal as a resource for searching and visualizing curated nucleomics data. Nat Commun 13, 2365. 10.1038/s41467-022-29697-4.
35676475	Emerson, D.J., Zhao, P.A., Cook, A.L., Barnett, R.J., Klein, K.N., Saulebekova, D., Ge, C., Zhou, L., Simandi, Z., Minsk, M.K., et al. (2022). Cohesin-mediated loop anchors confine the locations of human replication origins. Nature 606, 812-819. 10.1038/s41586-022-04803-0.

35817938	Boninsegna, L., Yildirim, A., Polles, G., Zhan, Y., Quinodoz, S.A., Finn, E.H., Guttman, M., Zhou, X.J., and Alber, F. (2022). Integrative genome modeling platform reveals essentiality of rare contact events in 3D genome organizations. Nat Methods 19, 938-949. 10.1038/s41592-022-01527-x.
35860407	Sengupta, K., Denkiewicz, M., Chilinski, M., Szczepinska, T., Mollah, A.F., Korsak, S., D'Souza, R., Ruan, Y., and Plewczynski, D. (2022). Multi-scale phase separation by explosive percolation with single-chromatin loop resolution. Comput Struct Biotechnol J 20, 3591-3603. 10.1016/j.csbj.2022.06.063.
35864166	Li, D., Harrison, J.K., Purushotham, D., and Wang, T. (2022). Exploring genomic data coupled with 3D chromatin structures using the WashU Epigenome Browser. Nat Methods 19, 909-910. 10.1038/s41592-022-01550-y.
35864167	Zhu, X., Zhang, Y., Wang, Y., Tian, D., Belmont, A.S., Swedlow, J.R., and Ma, J. (2022). Nucleome Browser: an integrative and multimodal data navigation platform for 4D Nucleome. Nat Methods 19, 911-913. 10.1038/s41592-022-01559-3.
36746940	Choudhary, M.N.K., Quaid, K., Xing, X., Schmidt, H., and Wang, T. (2023). Widespread contribution of transposable elements to the rewiring of mammalian 3D genomes. Nat Commun 14, 634. 10.1038/s41467-023-36364-9.
37336949	Beagrie, R.A., Thieme, C.J., Annunziatella, C., Baugher, C., Zhang, Y., Schueler, M., Kukalev, A., Kempfer, R., Chiariello, A.M., Bianco, S., et al. (2023). Multiplex-GAM: genome-wide identification of chromatin contacts yields insights overlooked by Hi-C. Nat Methods 20, 1037-1047. 10.1038/s41592-023-01903-1.
37580627	Yildirim, A., Hua, N., Boninsegna, L., Zhan, Y., Polles, G., Gong, K., Hao, S., Li, W., Zhou, X.J., and Alber, F. (2023). Evaluating the role of the nuclear microenvironment in gene function by population-based modeling. Nat Struct Mol Biol 30, 1193-1206. 10.1038/s41594-023-01036-1.
37845234	Calandrelli, R., Wen, X., Charles Richard, J.L., Luo, Z., Nguyen, T.C., Chen, C.J., Qi, Z., Xue, S., Chen, W., Yan, Z., et al. (2023). Genome-wide analysis of the interplay between chromatin-associated RNA and 3D genome organization in human cells. Nat Commun 14, 6519. 10.1038/s41467-023-42274-7.
37935964	Janssens, D.H., Greene, J.E., Wu, S.J., Codomo, C.A., Minot, S.S., Furlan, S.N., Ahmad, K., and Henikoff, S. (2024). Scalable single-cell profiling of chromatin modifications with sciCUT&Tag. Nat Protoc 19, 83-112. 10.1038/s41596-023-00905-9.
38538789	Wen, X., Luo, Z., Zhao, W., Calandrelli, R., Nguyen, T.C., Wan, X., Charles Richard, J.L., and Zhong, S. (2024). Single-cell multiplex chromatin and RNA interactions in ageing human brain. Nature 628, 648-656. 10.1038/s41586-024-07239-w.
38589516	Xiong, K., Zhang, R., and Ma, J. (2024). scGHOST: identifying single-cell 3D genome subcompartments. Nat Methods 21, 814-822. 10.1038/s41592-024-02230-9.
39271748	Kumar, P., Gholamalamdari, O., Zhang, Y., Zhang, L., Vertii, A., van Schaik, T., Peric-Hupkes, D., Sasaki, T., Gilbert, D.M., van Steensel, B., et al. (2024). Nucleolus and centromere Tyramide Signal Amplification-Seq reveals variable localization of heterochromatin in different cell types. Commun Biol 7, 1135. 10.1038/s42003-024-06838-7.
39681766	Yu, M., Zemke, N.R., Chen, Z., Juric, I., Hu, R., Raviram, R., Abnousi, A., Fang, R., Zhang, Y., Gorkin, D.U., et al. (2025). Integrative analysis of the 3D genome and epigenome in mouse embryonic tissues. Nat Struct Mol Biol 32, 479-490. 10.1038/s41594-024-01431-2.
39948644	Zhan, Y., Yildirim, A., Boninsegna, L., and Alber, F. (2025). Unveiling the role of chromosome structure morphology on gene function through chromosome conformation analysis. Genome Biol 26, 30. 10.1186/s13059-024-03472-8.
40279158	Gholamalamdari, O., van Schaik, T., Wang, Y., Kumar, P., Zhang, L., Zhang, Y., Gonzalez, G.A.H., Vouzas, A.E., Zhao, P.A., Gilbert, D.M., et al. (2025). Major nuclear locales define nuclear genome organization and function beyond A and B compartments. Elife 13. 10.7554/eLife.99116.
40329044	Irastorza-Azcarate, I., Kukalev, A., Kempfer, R., Thieme, C.J., Mastrobuoni, G., Markowski, J., Loof, G., Sparks, T.M., Brookes, E., Natarajan, K.N., et al. (2025). Extensive folding variability between homologous chromosomes in mammalian cells. Mol Syst Biol 21, 735-775. 10.1038/s44320-025-00107-3.

Supplementary Figure 1

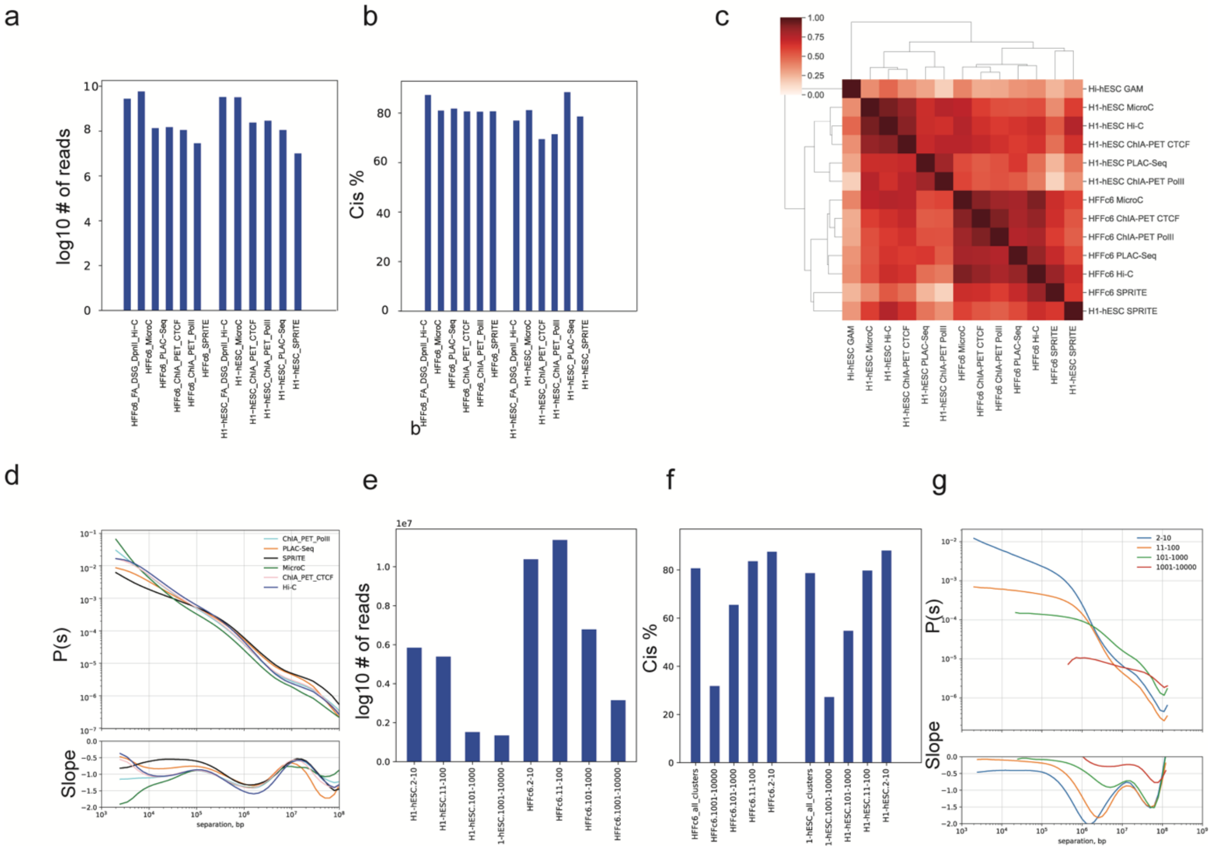

Legend Supplementary Figure 1

Quantitative metrics of data obtained with different chromatin interaction mapping methods

a. The number of reads obtained for Hi-C, Micro-C, ChIA PET, PLAC Seq, SPRITE datasets

b. The percentage of *cis* contacts for data obtained with each method indicated.

c. HiCRep {Yardimci, 2019 #1896} correlations of insulation profiles obtained with Hi-C, Micro-C, ChIA PET, PLAC Seq, GAM, and SPRITE

d. *P*(*s*) plot showing distance dependent contact probability of interactions detected with all protocols applied to HFFc6 cells (top). Derivative of the *P*(*s*) plots shown in panel d (bottom).

e. The number of fragments in each SPRITE cluster. Cluster sizes are 2-10, 11-100, 101-1000, 1001-10000 fragments.

f. The percentage of *cis* contacts in SPRITE clusters of indicated cluster size, for HFFc6 and H1-hESC cells.

g. *P*(*s*) plot showing distance dependent contact probability of interactions detected with different SPRITE cluster sizes for HFFc6 cells (top). Derivative of the *P*(*s*) plots shown in the bottom panel.

Supplementary Figure 2

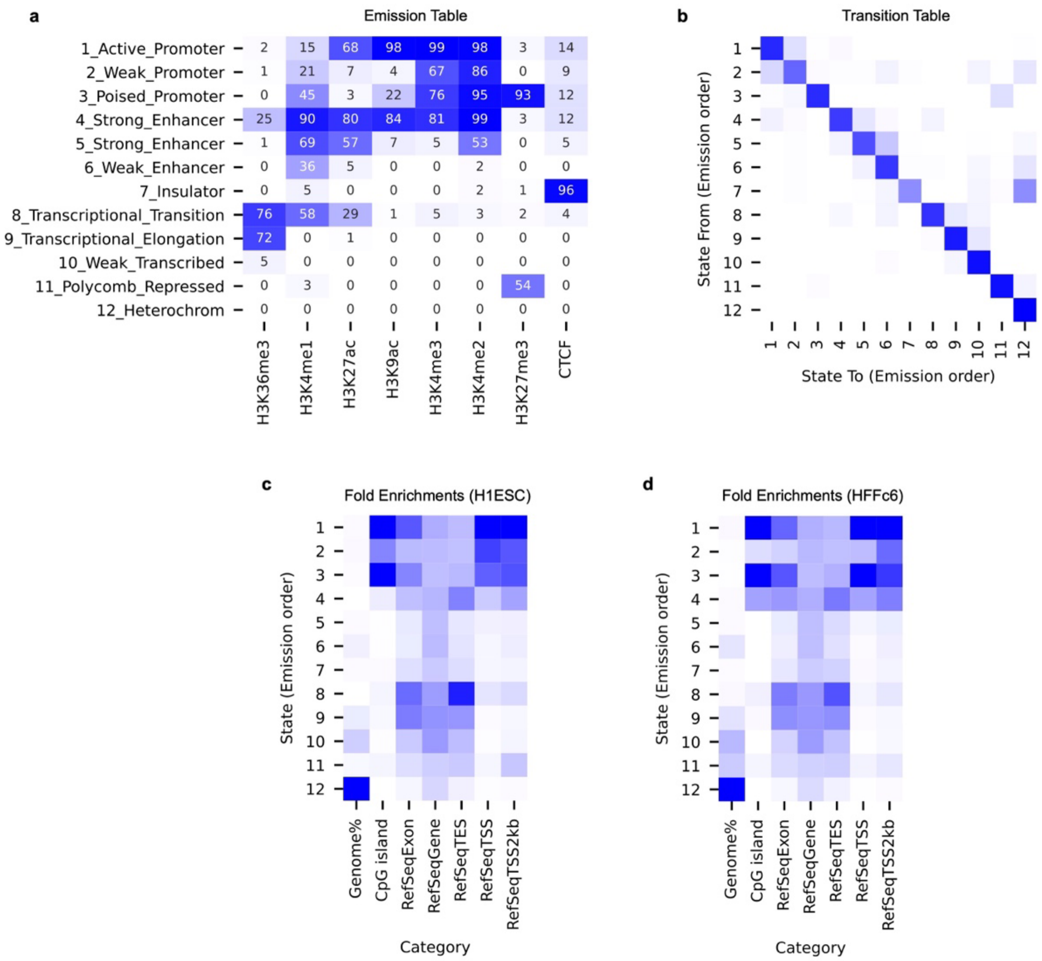

Legend Supplementary Figure 2

Consensus ChromHMM segmentation in H1-hESC and HFFc6.

a. Heatmap of emission parameters for the ChromHMM model.

b. Heatmap of transition parameters for the ChromHMM model.

c-d. Relative genomic coverage of each chromatin state and fold enrichment of chromatin states for selected genomic annotations in H1-hESC (c) and HFFc6 (d). TSS, transcription start site; TES, transcription end site.

Supplementary Figure 3

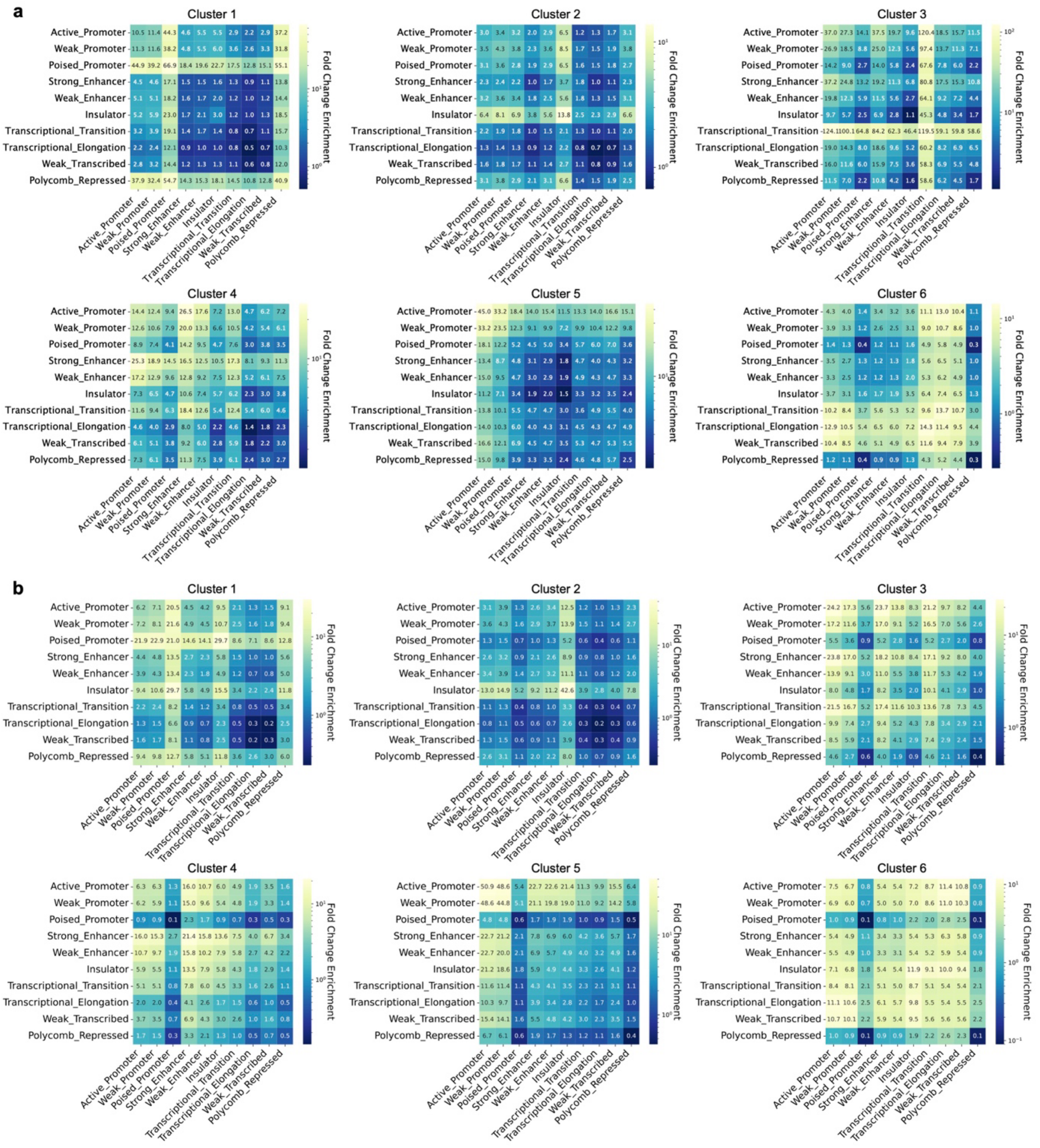

Legend Supplementary Figure 3

Fold-enrichment scores of ChromHMM states for different loop clusters revealed by the UMAP in H1-hESC (a) and HFFc6 (b).

Supplementary Figure 4

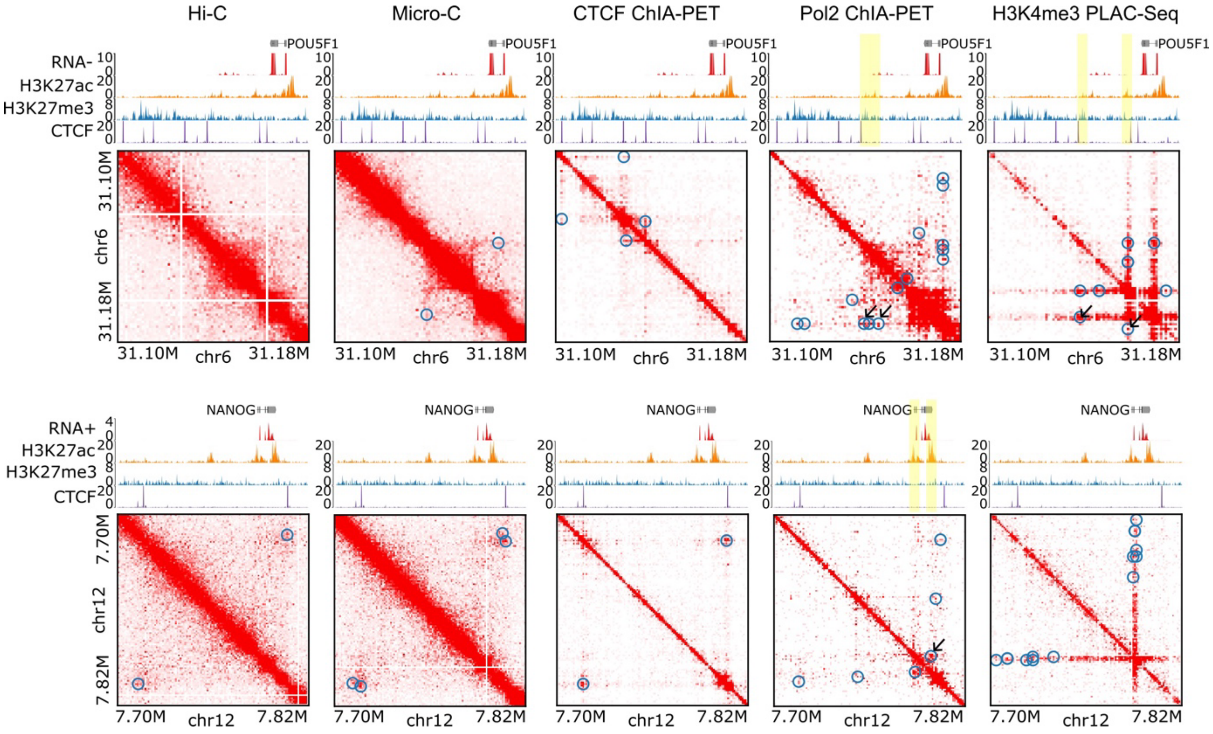

Legend Supplementary Figure 4

Two additional examples illustrating differences among platforms in detecting transcription-related loops.

Contact maps are plotted at 1 kb resolution, with chromatin loops detected by each platform marked by blue circles. Loops linking the indicated genes (*POU5F1* in the first example and *NANOG* in the second) to distal enhancers are indicated by black arrows, and the interacting distal enhancers are highlighted with yellow-shaded bars.

Supplementary Figure 5

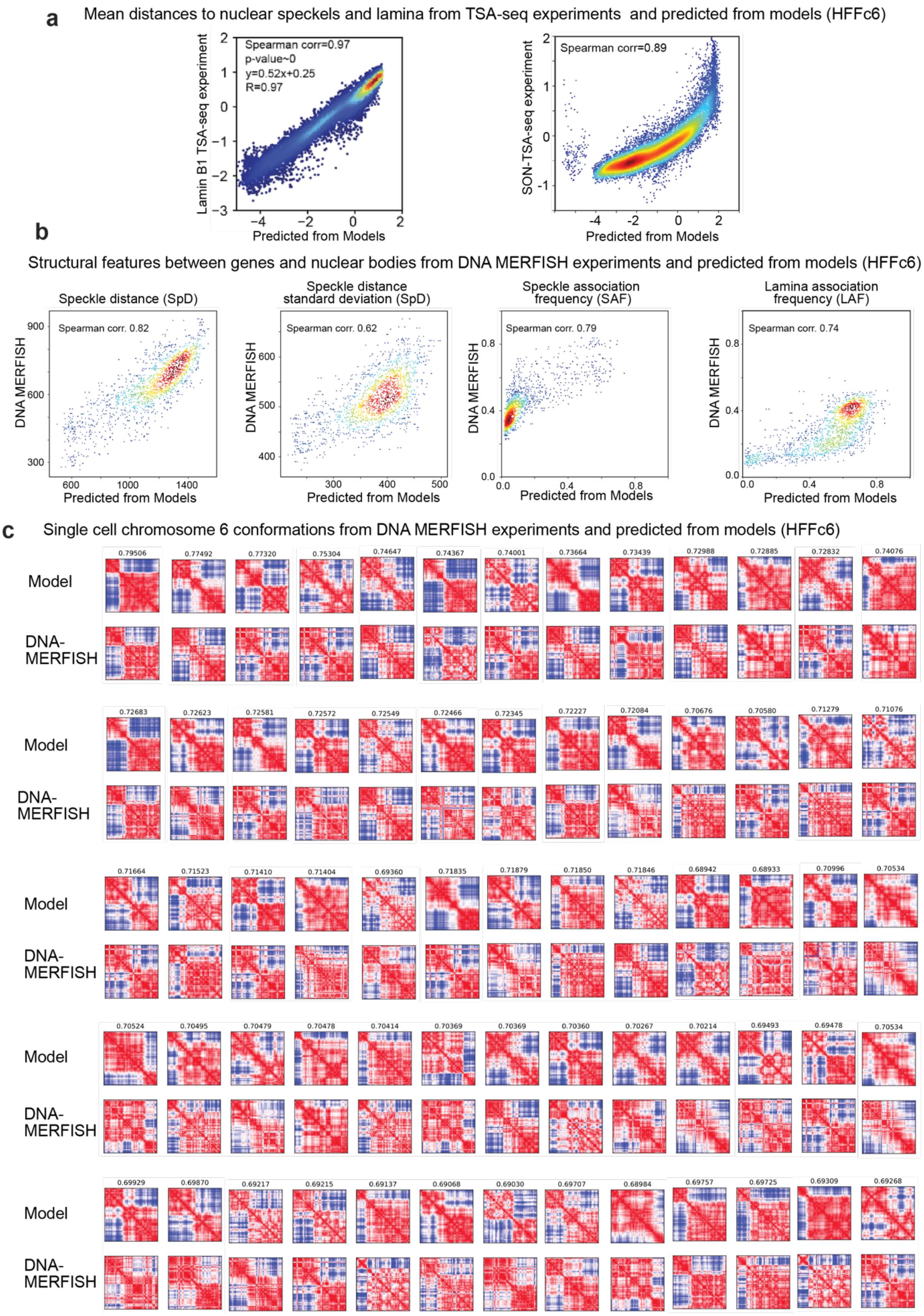

Legend Supplementary Figure 5

Assessment of 3D genome structure models against independent experimental data.

Assessment of genome structure models with independent experimental data.

a. Genome-wide correlation of TSA-seq data from experiment and predicted from our models (Left, Lamin B1 TSA-seq {Gholamalamdari, 2024 #1923}. Right, SON TSA-seq {Zhang, 2021 #1892}.

b. Genome-wide correlation of structure features between genome structures from DNA-MERFISH chromosome tracing experiments {Su, 2020 #1915} and predictions from our models. (From left to right, mean speckle distance (SpD), standard deviation of mean speckle distance in structure population, Speckle association frequency (SAF), Lamina association frequency (LAF)). **c.** Comparison of single cell distance matrices of chromosome 6 from simulated models (Top panel) and DNA-MERFISH imaging data {Su, 2020 #1915} (Bottom panel). Numbers above the distance matrices represent Pearson correlations between distance matrices from models and experiment.

Supplementary Figure 6

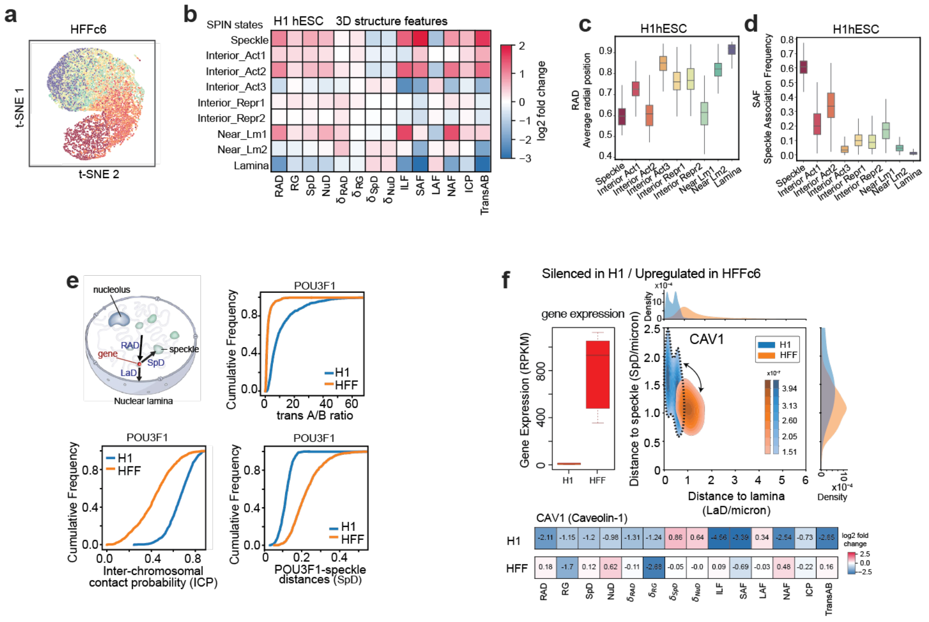

Legend Supplementary Figure 6

Genome-structure-function relationship

a. tSNE projections of structure feature vectors for chromatin in different SPIN states in HFFc6. (Color scheme of SPIN states defined as in panel c).

b. Log-fold enrichment of 14 3D structural features for chromatin in different SPIN states calculated from the population of models in H1 h-ESC cells.

c. Distributions of normalized radial positions for chromatin in different SPIN states calculated from a population of 3D structures of H1-hESC genomes.

d. Distributions of SAF values for chromatin in different SPIN states calculated from a population of 3D structures of H1-hESC genomes.

e. (Top left panel) Schematic view of structural features in single cell models. RAD: radial position, LaD, Distance to the nuclear envelope, SpD: Distance to the closest speckle. (Remaining panels) Cumulative distributions from single cell structure features of POU3F1 gene in the simulated cell population for Hffc6 and H1 h-ESC cells (SpD: speckle distance, ICP: inter-chromosomal contact probability, transAB ratio).

f. (Top left panel) Gene expression levels of CAV1 in H1-hESC and HFFc6 cells. (Top right panel) distribution of joint speckle distance and lamina distance for CAV1 genes in 1000 single cell models. (Lower panels) log-fold enrichment of 14 structure features for CAV1 gene in H1ESC and HFFc6 cells.

Supplementalry Figure 7

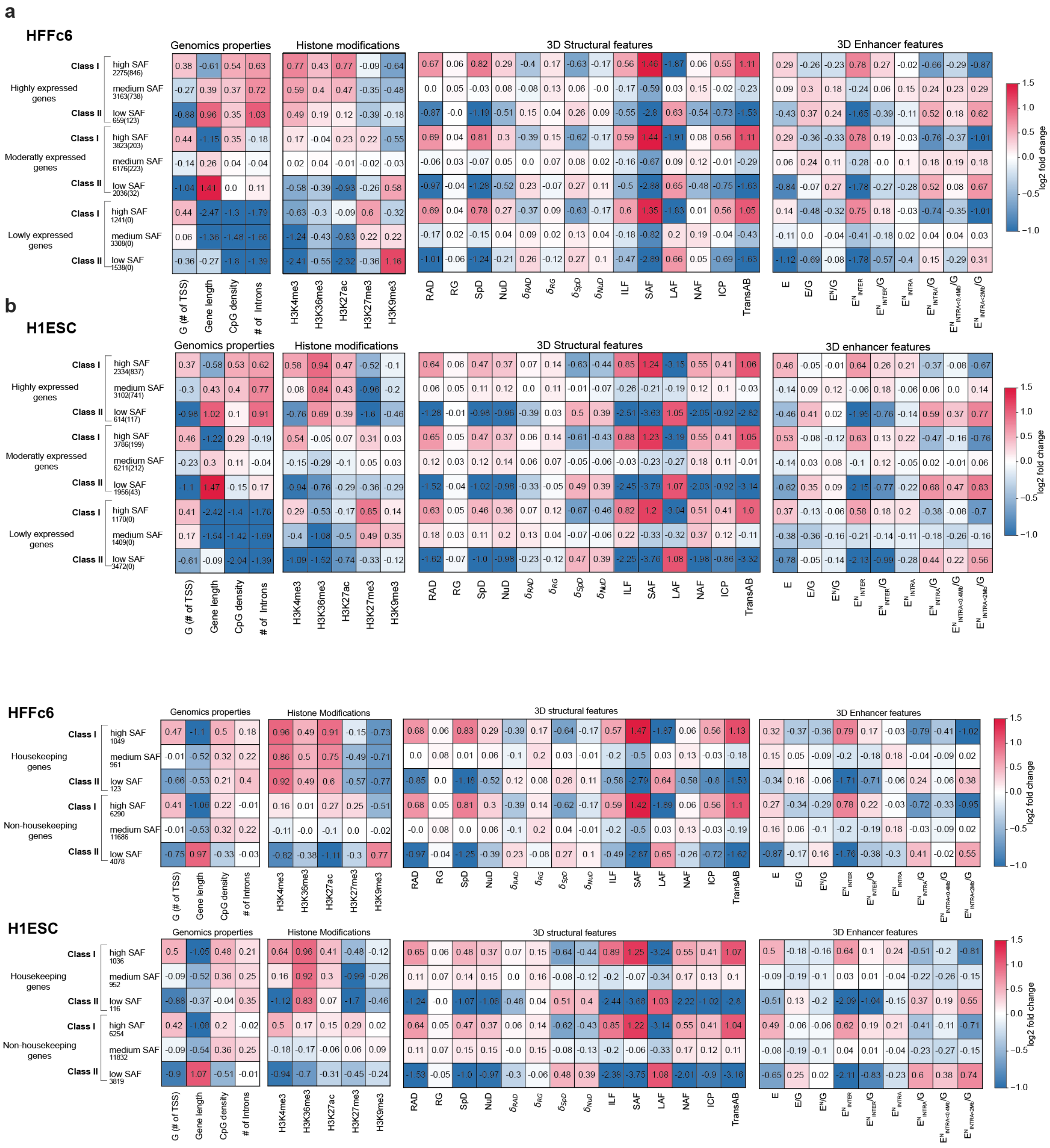

Legend Supplementary Figure 7

Enrichment plots for structural and functional features of genes.

a. Log-fold enrichment for genomic properties (calculated within a 200 kb region), histone modifications (within +/- 10 kb of TSS), 3D structure features, and 3D spatial enhancer densities at each TSS (Methods) for genes stratified by their speckle association frequencies in HFFc6. Details of spatial enhancer densities are defined in Methods.

b. Same as in panel **a** but for H1-hESC cells.

c. Same analysis for HFFc6 cells as in panel **a**, but now genes are stratified into housekeeping genes and non-housekeeping genes.

d. Same as in panel **c**, but for H1-hESC cells.

Supplementary Figure 8

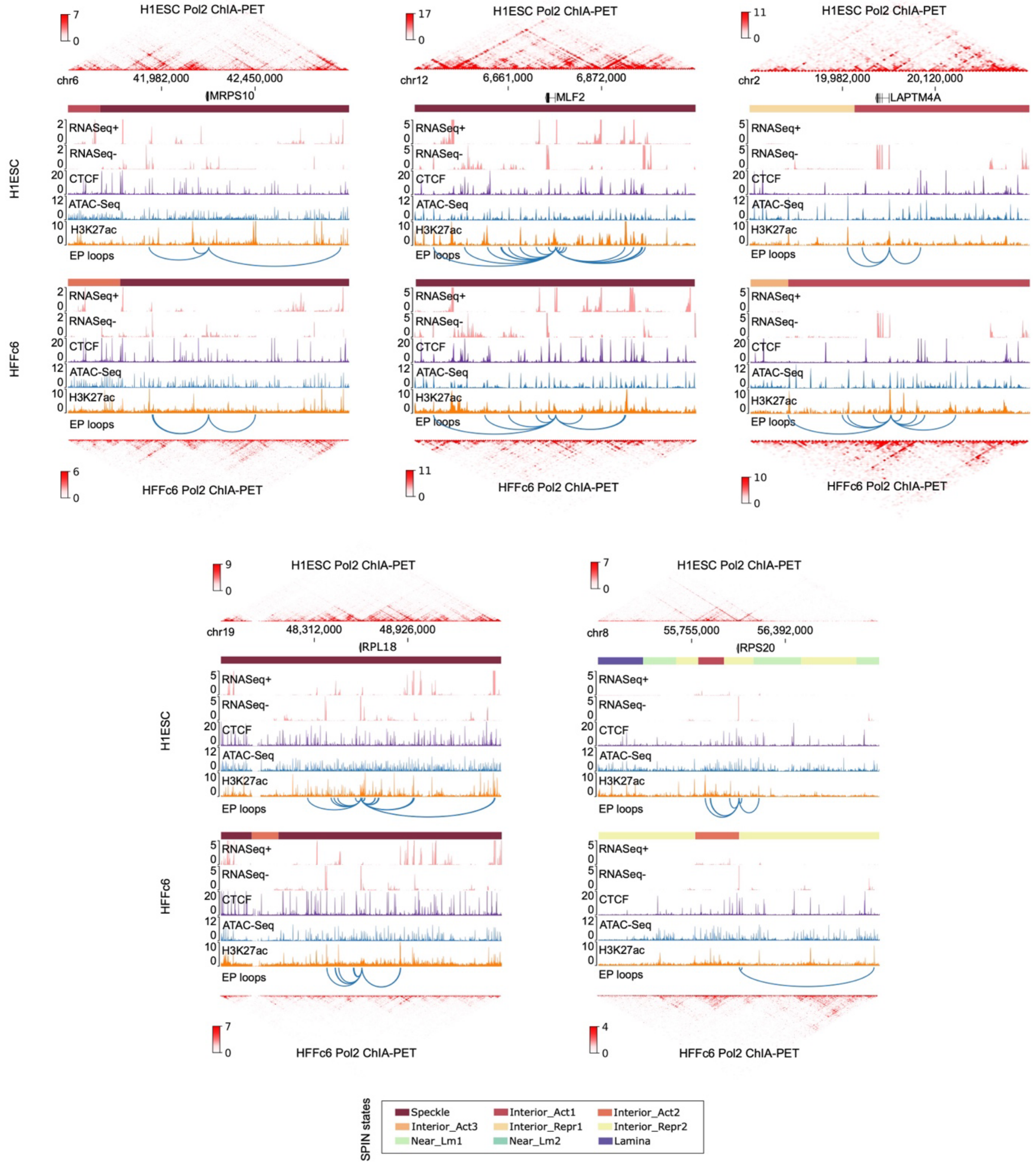

Legend Supplementary Figure 8

Example genome browser views showing cell-type-specific enhancer–promoter interactions of housekeeping genes.

Blue arcs represent chromatin loops linking the promoters of the indicated housekeeping genes to distal enhancers.

Supplementalry Figure 9

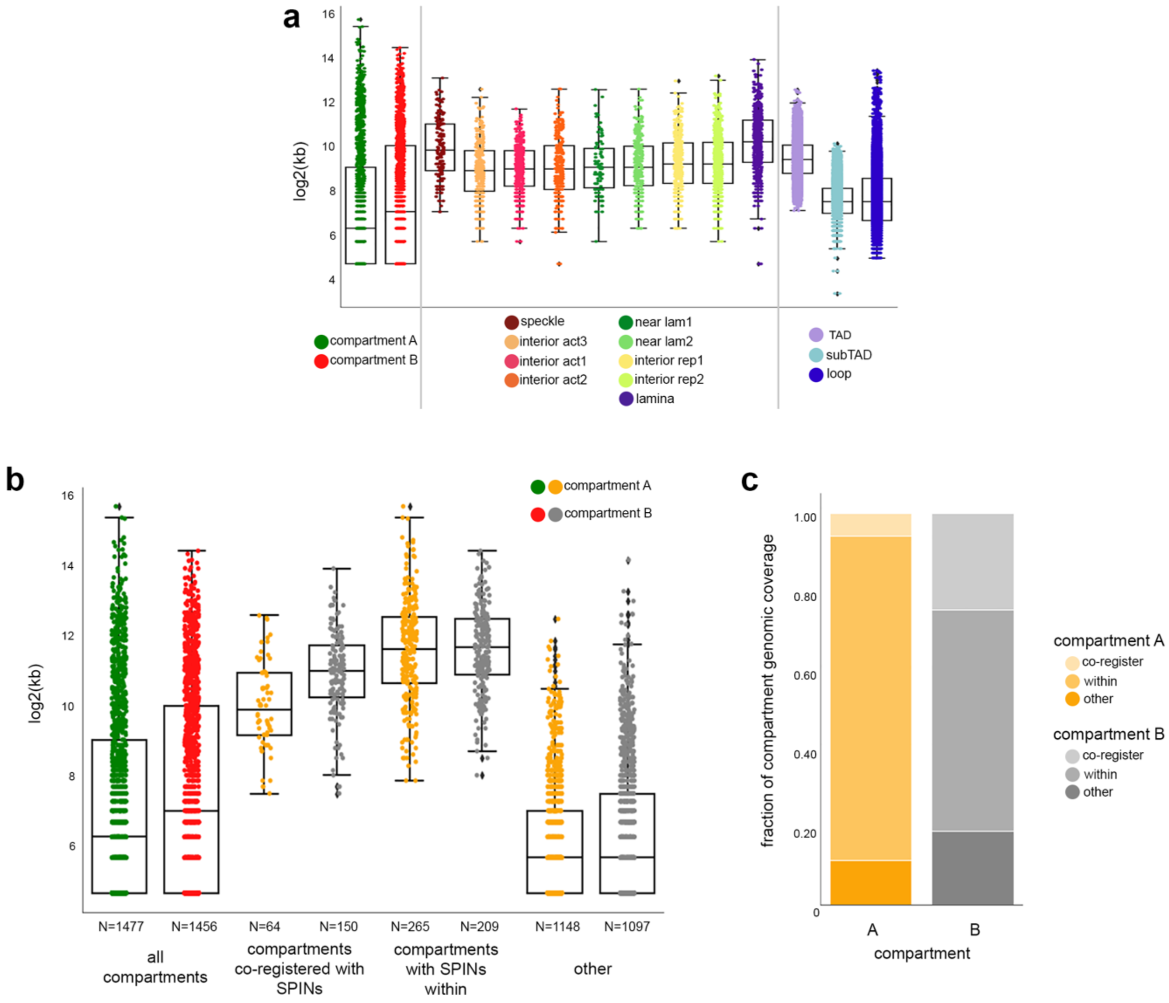

Legend Supplementary Figure 9

Compartments and SPIN comparison.

a. Size comparison of genome folding features in H1-hESCs. All regions plotted by size of their genomic coordinates, box plot shows 25th, 50th, and 75th quartiles, whiskers show minimum and maximum values with outliers annotated.

b. Genomic size of A/B compartments stratified by SPIN alignment in H1-hESCs. Total compartment A (green; N=1477) and total compartment B (red ; N=1456) were stratified by those that co-register with a SPIN, fully encompass or contain a SPIN within, or other, including nested within a SPIN and no SPIN intersection. **c.** Genomic coverage of A/B compartments stratified by SPIN alignment.

Supplementary Figure 10

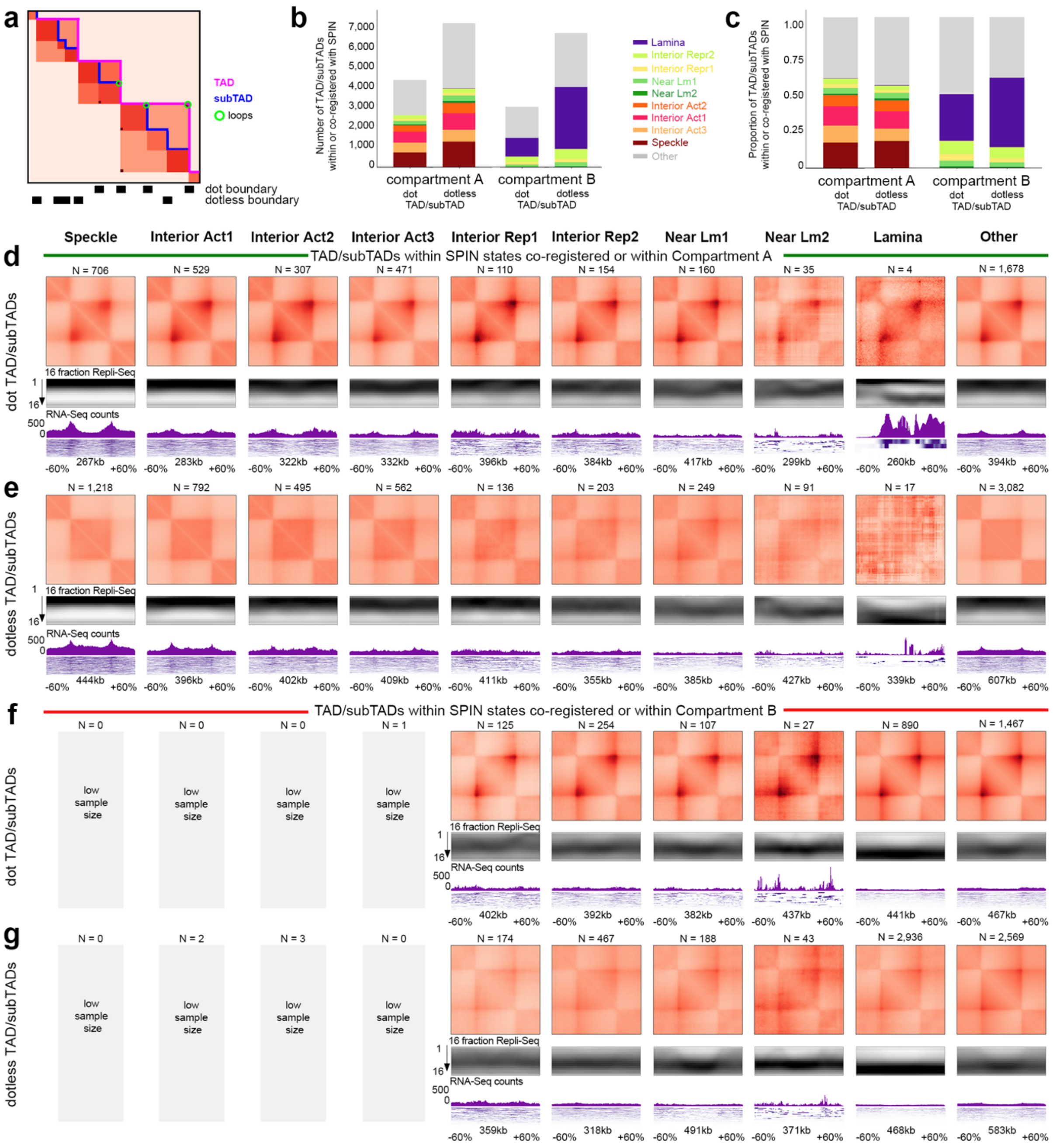

Legend Supplementary Figure 10

Compartment, SPIN, and TAD integration with replication timing, RNA-seq, and nascent transcripts from iMargi.

a. Schematic depicting TAD (pink) and subTADs (blue) domains and loops (green circle). Dot domains contain a loop at the domain apex and dot boundaries. Dotless domains do not contain a loop at the apex and thus only dotless boundaries.

b.,c. Dot and dotless TAD/subTAD domains that are within or co- register with a SPIN state contained within or co-registering with A/B compartments. **b.** Number of and **c.** proportion of SPINs stratified by A/B compartment and presence of corner-dot TAD/subTAD domains.

d.-g. Averaged Hi-C, replication timing (16 fraction Repli-seq), mRNA levels (RNA-seq) for H1-hESCs at dot and dotless TAD/subTAD domains co-registered or co- localized within all SPIN states (columns 1-9) and TAD/subTAD domains with other SPIN alignment (column 10) either co-registered or co-localized within A/B compartments. All genomics data is plotted as the average signal across all genomic intervals representing domains in a particular column. TAD/subTAD genomic intervals of (TAD/subTAD genomic interval +/- flanks of 60% of the size of the genomic interval) are stretched laterally to scale by size before average signal is computed. **d.** Dot and **e.** dotless TAD/subTAD domain co- registered or within a SPIN and co-registered or within compartment A and **f.** Dot and **g.** dotless TAD/subTAD domain co-registered or within a SPIN and co-registered or within compartment B. Tracks show pileups in H1-hESC for Hi-C Aggregate-Peak-Analysis (APA), 16 fraction Repli-seq, median RNA-seq signal, condensed RNA-seq reads.

Supplementary Figure 11

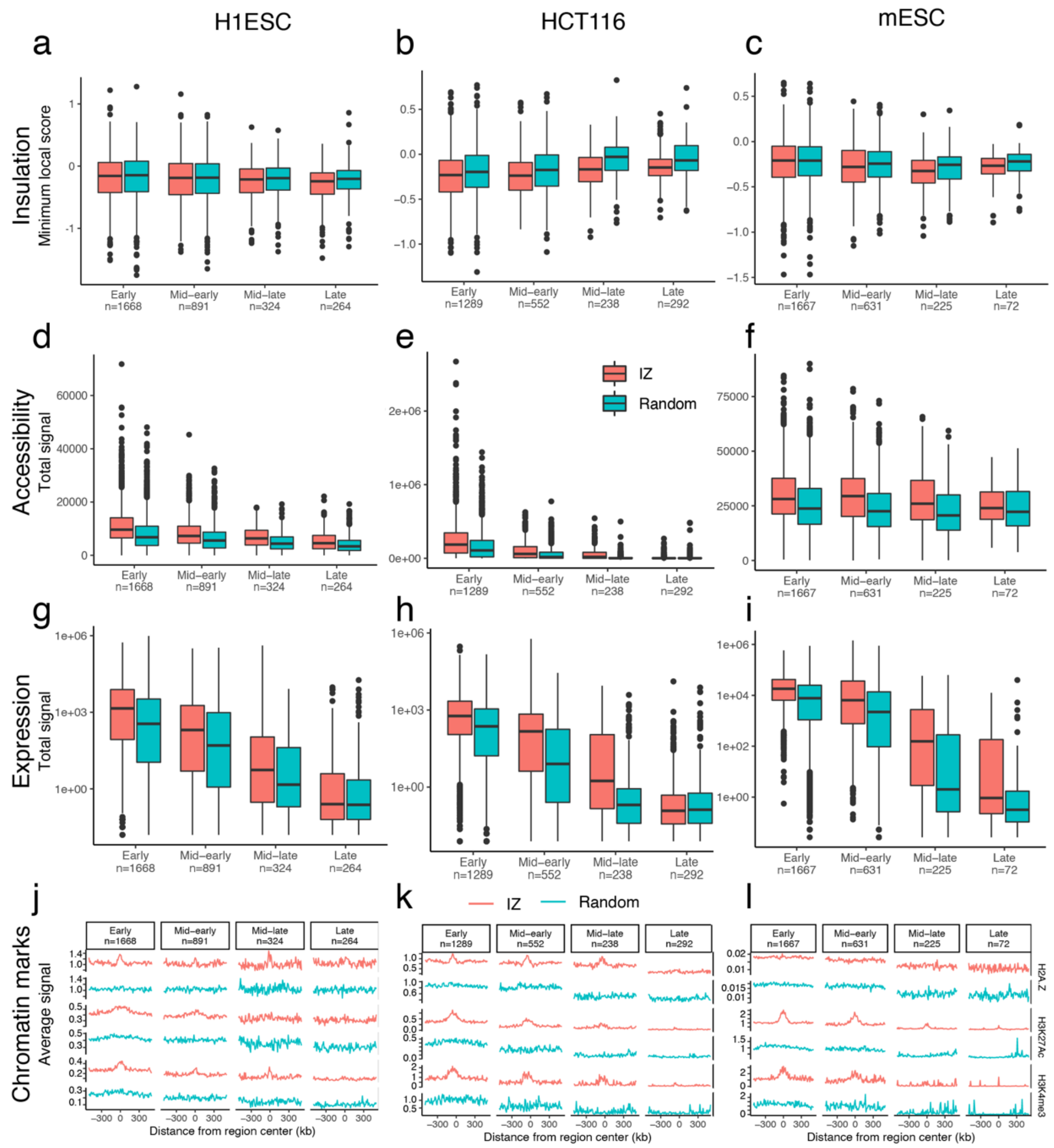

Legend Supplementary Figure 11

Comparison of IZ properties across cell lines.

a.-c. Boxplots showing the minimum diamond insulation score at IZs in H1-hESC (**a**), HCT116 (**b**) and mESC (**b**).

d.-f. Boxplots showing the total chromatin accessibility at IZs in H1-hESC (**d**), HCT116 (**e**), and mESC (**f**).

g.-i. Boxplots showing the total RNA signal in H1-hESC (**g**), HCT116 (**h**), and mESC (**i**).

j.-l. Average histone marks signal in 1Mb regions centered on IZs in H1-hESC (**j**), HCT116 (**k**), and mESC (**l**).

Boxplots represent the median and interquartile range (IQR); whiskers mark 1.5x the IQR; data beyond 1.5x the IQR are plotted as individual points.

Supplementary Figure 12

a

Application	general nuclear organization (chromosome, compartment level), in cell lines and cultured cells	general nuclear organization in small cell populations, up to tens of thousands of cells	Analysis in single cells	analysis of mixed samples, e.g., compex tissues	Genome rearrangement analysis	Sub kb-resolution analysis	detection of looping interactions between cCREs genome-wide	detection of looping interactions between cCREs in specific loci of up to several Mb
**Assay**								
**Hi-C**	***	**	-	*	***	***	**	*
**Micro-C**	***	*	-	*	***	***	**	*
**Chia-PET**	*	*	-	*	*	**	***	**
**PLAC-seq / Hi-ChIP**	*	*	-	*	*	**	***	**
**GAM**	***	***	***	***	*	*	*	*
**Capture-Micro-C**	*	*	-	*	**	***	***	***
**Capture-C**	*	*	-	*	**	***	***	***
**Single cell Hi-C**	**	***	***	***	*	*	*	*
**Single cell HiChip/ChiaPet**	**	**	***	***	*	*	*	*

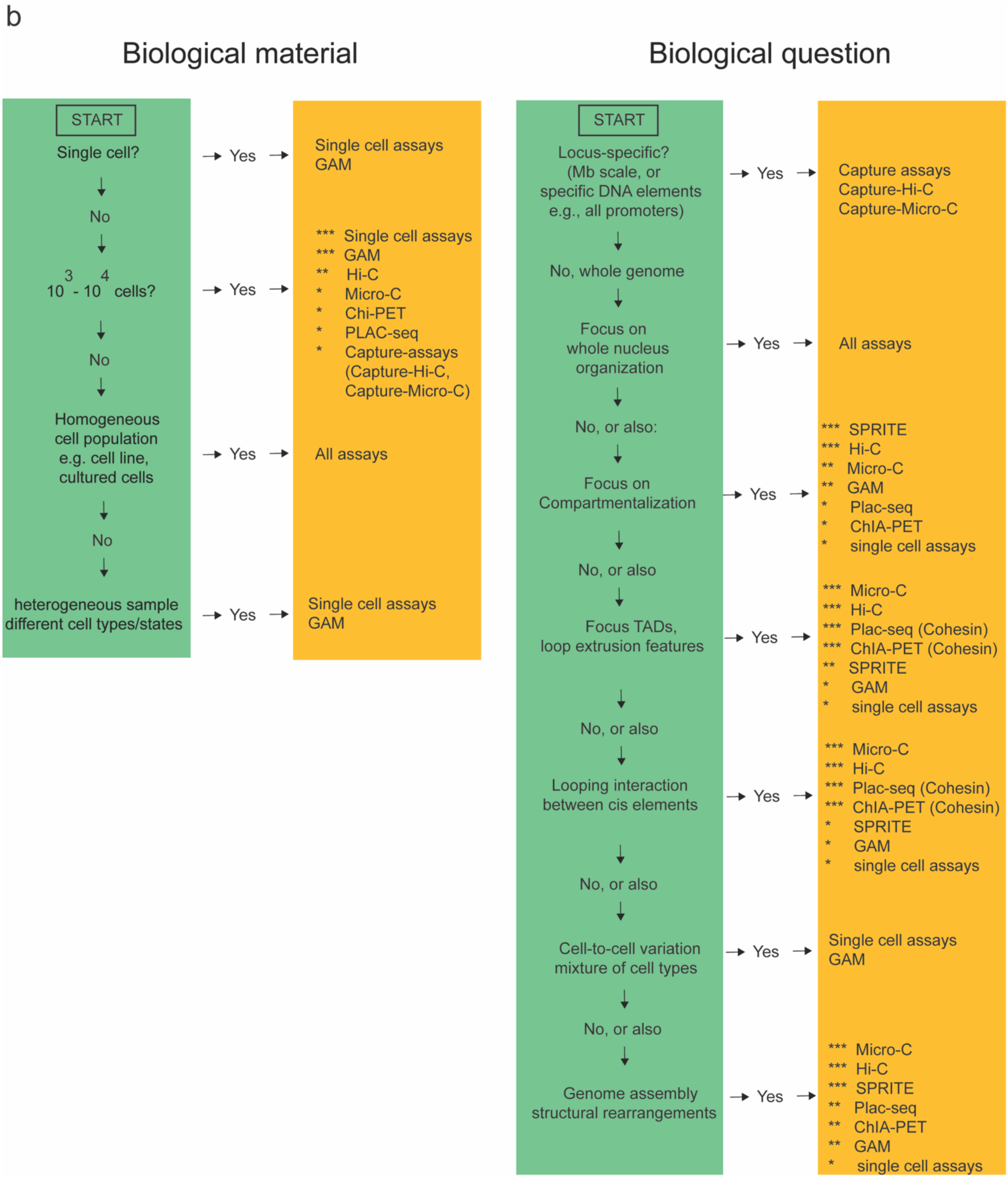

Legend Supplementary Figure 12

User guide and decision trees for assay usage

a. Table with recommendations for usage of indicated assays for indicated applications
b. Decision trees for assay selection focused starting biological material (left), and biological question (right).

***: well suited; **: can be used; * less suited, -: not applicable.

Supplementary Figure 13

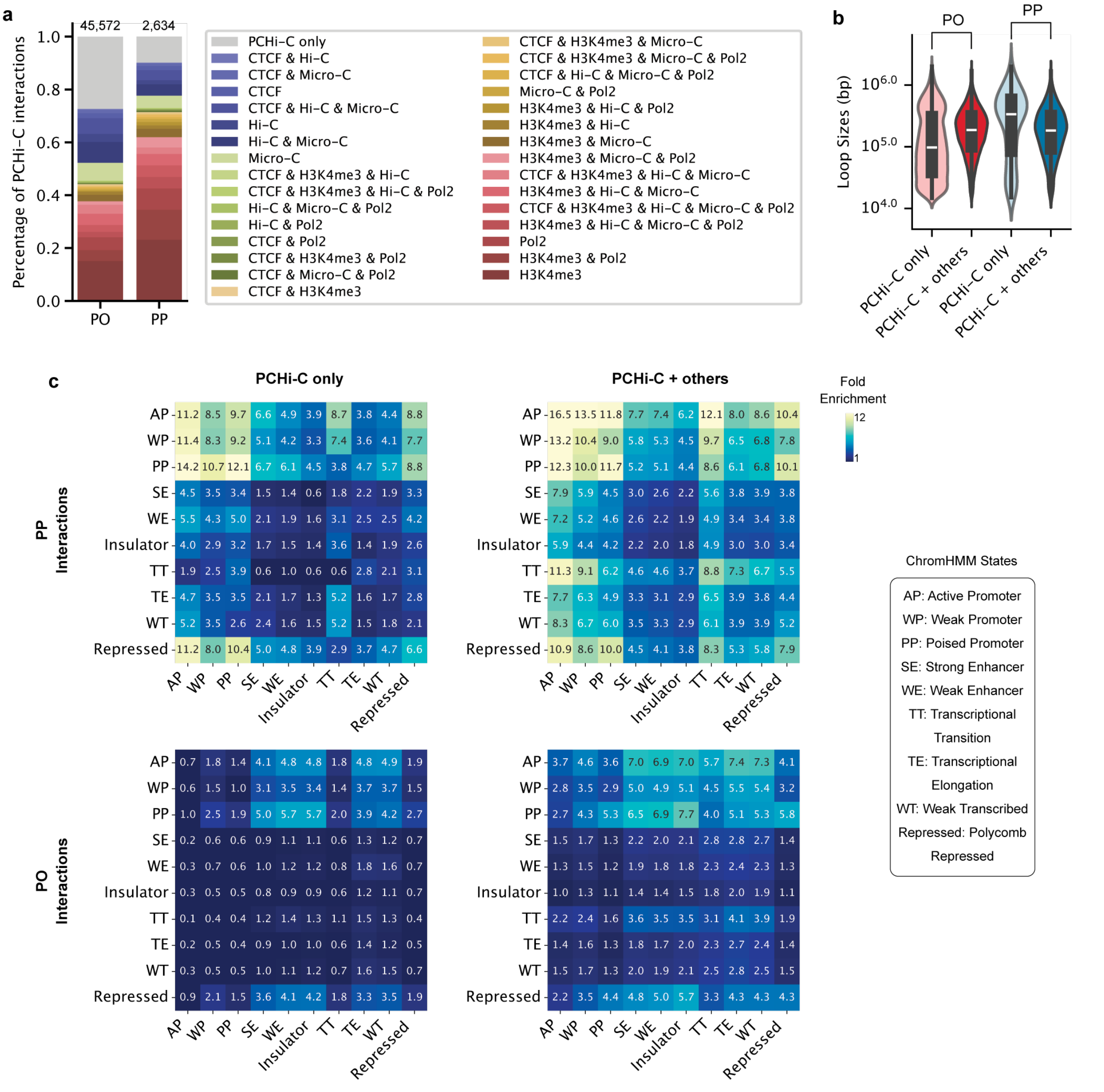

Legend Supplementary Figure 13

Comparison of Promoter Capture Hi-C Interactions with Loops Identified by Other 3D Genomic Methods. A published Promoter Capture Hi-C (PCHi-C) dataset in H1-hESC cells was used for this analysis {Jung, 2019 #2049}. Promoter–promoter (PP) and promoter–other (PO) interactions were analyzed separately.

a. Percentage of PP and PO PCHi-C interactions overlapping with loops identified by various combinations of other 3D genomic methods. The number on top of each bar indicates the total interaction number for each category.

b. Comparison of genomic distances between interacting anchors (i.e., loop sizes) for interactions unique to PCHi-C versus those also supported by other methods.

c. Fold-enrichment of ChromHMM states at loop anchors for interactions unique to PCHi-C or shared with other 3D genomic methods.

Supplementary Note 1

Publicly available datasets used in this study, organized by section of the paper

1. Methods and data for benchmarking section

Datasets

Cell Type	Experimental Type	Data Type	Data Source/Download Link	Note
H1-ESC	Hi-C	Contact matrix	4DN (4DNFI82R42AD)	
	Micro-C	Contact matrix	4DN (4DNFI9GMP2J8)	
	CTCF ChIA-PET	Contact matrix	4DN (4DNFINMHXGVQ)	
	RNAPII ChIA-PET	Contact matrix	4DN (4DNFIO8YJ5JA)	
	H3K4me3 PLAC-Seq	Contact matrix	4DN (4DNFICOGAKW2)	
	DNA SPRITE	Contact matrix	4DN (4DNFITX6WCRT)	
		Clusters	4DN (4DNFIV3PDS5F, 4DNFIIY1TXUZ)	
	GAM	Contact matrix at 25kb	4DNESVAMUDHA	
	CTCF ChIP-Seq	Signal (Bigwig)	https://epigenome.wustl.edu/epimap/data/observed/FINAL_CTCF_BSS00478.sub_VS_FINAL_WCE_BSS00478.pval.signal.bedgraph.gz.bigWig	ChromHMM Input
	H3K27ac ChIP-Seq	Signal (Bigwig)	https://epigenome.wustl.edu/epimap/data/observed/FINAL_H3K27ac_BSS00478.sub_VS_FINAL_WCE_BSS00478.pval.signal.bedgraph.gz.bigWig	
	H3K27me3 ChIP-Seq	Signal (Bigwig)	https://epigenome.wustl.edu/epimap/data/observed/FINAL_H3K27me3_BSS00478.sub_VS_FINAL_WCE_BSS00478.pval.signal.bedgraph.gz.bigWig	
	H3K36me3 ChIP-Seq	Signal (Bigwig)	https://epigenome.wustl.edu/epimap/data/observed/FINAL_H3K36me3_BSS00478.sub_VS_FINAL_WCE_BSS00478.pval.signal.bedgraph.gz.bigWig	

H3K4me1 ChIP-Seq	Signal (Bigwig)	https://epigenome.wustl.edu/epimap/data/observed/FINAL_H3K4me1_BSS00478.sub_VS_FINAL_WCE_BSS00478.pval.signal.bedgraph.gz.bigWig	
H3K9ac ChIP-Seq	Signal (Bigwig)	https://epigenome.wustl.edu/epimap/data/observed/FINAL_H3K9ac_BSS00478.sub_VS_FINAL_WCE_BSS00478.pval.signal.bedgraph.gz.bigWig	
H3K4me3 ChIP-Seq	Signal (Bigwig)	https://epigenome.wustl.edu/epimap/data/observed/FINAL_H3K4me3_BSS00478.sub_VS_FINAL_WCE_BSS00478.pval.signal.bedgraph.gz.bigWig	
H3K4me2 ChIP-Seq	Signal (Bigwig)	https://epigenome.wustl.edu/epimap/data/observed/FINAL_H3K4me2_BSS00478.sub_VS_FINAL_WCE_BSS00478.pval.signal.bedgraph.gz.bigWig	
RNA-Seq	Signal (Bigwig)	ENCODE (ENCFF501KFP, ENCFF563OKS)	Visualization
CTCF ChIP-Seq	Signal (Bigwig)	ENCODE (ENCFF332TNJ)	
H3K27ac ChIP-Seq	Signal (Bigwig)	ENCODE (ENCFF103PND)	
H3K27me3 ChIP-Seq	Signal (Bigwig)	ENCODE (ENCFF502GXT)	
EZH2 ChIP-Seq	Signal (Bigwig)	ENCODE (ENCFF109KCQ)	
POLR2A ChIP-Seq	Signal (Bigwig)	ENCODE (ENCFF942TZX)	
CHD1 ChIP-Seq	Signal (Bigwig)	ENCODE (ENCFF597VKW)	
KDM4A ChIP-Seq	Signal (Bigwig)	ENCODE (ENCFF269CHA)	
PHF8 ChIP-Seq	Signal (Bigwig)	ENCODE (ENCFF059EBB)	
TAF1 ChIP-Seq	Signal (Bigwig)	ENCODE (ENCFF837BSZ)	
RAD21 ChIP-Seq	Signal (Bigwig)	ENCODE (ENCFF056GWP)	
KDM1A ChIP-Seq	Peaks	ENCODE (ENCFF759CSN)	Enrichment analysis
CBX8 ChIP-Seq	Peaks	ENCODE (ENCFF483UZG)	
EZH2 ChIP-Seq	Peaks	ENCODE (ENCFF414CAB)	
KDM4A ChIP-Seq	Peaks	ENCODE (ENCFF021QGZ)	
SP4 ChIP-Seq	Peaks	ENCODE (ENCFF257FUV)	
MAX ChIP-Seq	Peaks	ENCODE (ENCFF994IHO)	
POU5F1 ChIP-Seq	Peaks	ENCODE (ENCFF383EYO)	
CHD1 ChIP-Seq	Peaks	ENCODE (ENCFF731EYW)	
ZNF143 ChIP-Seq	Peaks	ENCODE (ENCFF235ROG)	
TAF1 ChIP-Seq	Peaks	ENCODE (ENCFF886BPR)	
TCF12 ChIP-Seq	Peaks	ENCODE (ENCFF959HJP)	

CHD7 ChIP-Seq	Peaks	ENCODE (ENCFF338IDU)
CEBPB ChIP-Seq	Peaks	ENCODE (ENCFF823KCM)
BRCA1 ChIP-Seq	Peaks	ENCODE (ENCFF721TNS)
MXI1 ChIP-Seq	Peaks	ENCODE (ENCFF727RNJ)
CTBP2 ChIP-Seq	Peaks	ENCODE (ENCFF501VJW)
SIRT6 ChIP-Seq	Peaks	ENCODE (ENCFF219XEX)
SUZ12 ChIP-Seq	Peaks	ENCODE (ENCFF225AMM)
EP300 ChIP-Seq	Peaks	ENCODE (ENCFF244VKF)
ZNF274 ChIP-Seq	Peaks	ENCODE (ENCFF718OGI)
HDAC6 ChIP-Seq	Peaks	ENCODE (ENCFF802HUU)
GABPA ChIP-Seq	Peaks	ENCODE (ENCFF308NZT)
POLR2A ChIP-Seq	Peaks	ENCODE (ENCFF322DAE)
GTF2F1 ChIP-Seq	Peaks	ENCODE (ENCFF138MYA)
MYC ChIP-Seq	Peaks	ENCODE (ENCFF049SMR)
NRF1 ChIP-Seq	Peaks	ENCODE (ENCFF414RES)
REST ChIP-Seq	Peaks	ENCODE (ENCFF738LQB)
JUN ChIP-Seq	Peaks	ENCODE (ENCFF821GUI)
HDAC2 ChIP-Seq	Peaks	ENCODE (ENCFF923TXH)
RXRA ChIP-Seq	Peaks	ENCODE (ENCFF745EBL)
CTCF ChIP-Seq	Peaks	ENCODE (ENCFF023LAA)
YY1 ChIP-Seq	Peaks	ENCODE (ENCFF376FVJ)
ATF3 ChIP-Seq	Peaks	ENCODE (ENCFF440FTA)
USF2 ChIP-Seq	Peaks	ENCODE (ENCFF346KIW)
BCL11A ChIP-Seq	Peaks	ENCODE (ENCFF847HXU)
JUND ChIP-Seq	Peaks	ENCODE (ENCFF287KKY)
USF1 ChIP-Seq	Peaks	ENCODE (ENCFF978MNS)
RNF2 ChIP-Seq	Peaks	ENCODE (ENCFF241UKW)
SP2 ChIP-Seq	Peaks	ENCODE (ENCFF309QRC)
SIX5 ChIP-Seq	Peaks	ENCODE (ENCFF384KWP)
CBX5 ChIP-Seq	Peaks	ENCODE (ENCFF218OXB)
ATF2 ChIP-Seq	Peaks	ENCODE (ENCFF352KLD)
RFX5 ChIP-Seq	Peaks	ENCODE (ENCFF142NQQ)
FOSL1 ChIP-Seq	Peaks	ENCODE (ENCFF428RHR)
NANOG ChIP-Seq	Peaks	ENCODE (ENCFF435DTC)
POLR2AphosphoS5 ChIP-Seq	Peaks	ENCODE (ENCFF872MKT)
BACH1 ChIP-Seq	Peaks	ENCODE (ENCFF749UPP)
E2F6 ChIP-Seq	Peaks	ENCODE (ENCFF174AVU)
CHD2 ChIP-Seq	Peaks	ENCODE (ENCFF726GBF)
RAD21 ChIP-Seq	Peaks	ENCODE (ENCFF883FUW)
SRF ChIP-Seq	Peaks	ENCODE (ENCFF648QJE)
EGR1 ChIP-Seq	Peaks	ENCODE (ENCFF100KKH)
TEAD4 ChIP-Seq	Peaks	ENCODE (ENCFF885PQR)
TBP ChIP-Seq	Peaks	ENCODE (ENCFF817TGF)

	CREB1 ChIP-Seq	Peaks	ENCODE (ENCFF978KGB)	
	MAFK ChIP-Seq	Peaks	ENCODE (ENCFF710YJK)	
	RBBP5 ChIP-Seq	Peaks	ENCODE (ENCFF610PCN)	
	SIN3A ChIP-Seq	Peaks	ENCODE (ENCFF432EYM)	
	SP1 ChIP-Seq	Peaks	ENCODE (ENCFF284JVS)	
	SAP30 ChIP-Seq	Peaks	ENCODE (ENCFF043HXQ)	
	KDM5A ChIP-Seq	Peaks	ENCODE (ENCFF471OCM)	
	ASH2L ChIP-Seq	Peaks	ENCODE (ENCFF693FGQ)	
HFFc6	Hi-C	Contact matrix	4DN (4DNFIAVXXO55)	
	Micro-C	Contact matrix	4DN (4DNFI9FVHJZQ)	
	CTCF ChIA-PET	Contact matrix	4DN (4DNFIG2ILS39)	
	RNAPII ChIA-PET	Contact matrix	4DN (4DNFIIASUSSX)	
	H3K4me3 PLAC-Seq	Contact matrix	4DN (4DNFI9REIU8H)	
	DNA SPRITE	Contact matrix	4DN (4DNFIP9HGF9M)	
		Clusters	4DN (4DNFIRXON7Z2)	
	CTCF ChIP-Seq	Signal (Bigwig)	https://epigenome.wustl.edu/epimap/data/observed/FINAL_CTCF_BSS00353.sub_VS_FINAL_WCE_BSS00353.pval.signal.bedgraph.gz.bigWig	ChromHMM Input
	H3K27ac ChIP-Seq	Signal (Bigwig)	https://epigenome.wustl.edu/epimap/data/observed/FINAL_H3K27ac_BSS00353.sub_VS_FINAL_WCE_BSS00353.pval.signal.bedgraph.gz.bigWig	
	H3K27me3 ChIP-Seq	Signal (Bigwig)	https://epigenome.wustl.edu/epimap/data/observed/FINAL_H3K27me3_BSS00353.sub_VS_FINAL_WCE_BSS00353.pval.signal.bedgraph.gz.bigWig	
	H3K36me3 ChIP-Seq	Signal (Bigwig)	https://epigenome.wustl.edu/epimap/data/observed/FINAL_H3K36me3_BSS00353.sub_VS_FINAL_WCE_BSS00353.pval.signal.bedgraph.gz.bigWig	
	H3K4me1 ChIP-Seq	Signal (Bigwig)	https://epigenome.wustl.edu/epimap/data/observed/FINAL_H3K4me1_BSS00353.sub_VS_FINAL_WCE_BSS00353.pval.signal.bedgraph.gz.bigWig	
	H3K9ac ChIP-Seq	Signal (Bigwig)	https://epigenome.wustl.edu/epimap/data/imputed/impute_BSS00353_H3K9ac.bigWig	

H3K4me3 ChIP-Seq	Signal (Bigwig)	https://epigenome.wustl.edu/epimap/data/observed/FINAL_H3K4me3_BSS00353.sub_VS_FINAL_WCE_BSS00353.pval.signal.bedgraph.gz.bigWig
H3K4me2 ChIP-Seq	Signal (Bigwig)	https://epigenome.wustl.edu/epimap/data/imputed/impute_BSS00353_H3K4me2.bigWig

GAM 4DN accession number: 4DNESVAMUDHA (replicates id 4DNBS46FF9D9, 4DNBSB45FJC1)
Software: bowtie2 (v.2.3.4.3), samtools (v.1.14), bedtools (v.2.30), fastq_screen (v.0.14.0), GEM-tools(v.1.315)

Data processing - Hi-C, Micro-C, ChIA-PET, PLAC-Seq and SPRITE
The link for Hi-C, Micro-C and ChIA-PET files can be found here:
https://data.4dnucleome.org/browse/?type=ExperimentSetReplicate&experimentset_type=replicate&experiments_in_set.biosample.biosource.organism.name=human&experiments_in_set.biosample.biosource_summary=HFFc6+%28Tier+1%29&experiments_in_set.biosample.biosource_summary=H1-hESC+%28Tier+1%29&experiments_in_set.experiment_categorizer.combined=Target%3A+RNA+Pol+II&experiments_in_set.experiment_categorizer.combined=Target%3A+CTCF+protein&experiments_in_set.experiment_categorizer.combined=Enzyme%3A+DpnII&experiments_in_set.experiment_categorizer.combined=Enzyme%3A+MNase&experiments_in_set.experiment_type.display_title=in+situ+Hi-C&experiments_in_set.experiment_type.display_title=DNA+SPRITE&experiments_in_set.experiment_type.display_title=Micro-C&experiments_in_set.experiment_type.display_title=PLAC-seq&experiments_in_set.experiment_type.display_title=in+situ+ChIA-PET.

Link for PLAC-Seq and SPRITE files can be found here:
https://data.4dnucleome.org/browse/?type=ExperimentSetReplicate&experimentset_type=replicate&experiments_in_set.biosample.biosource.organism.name=human&experiments_in_set.biosample.biosource_summary=H1-hESC+%28Tier+1%29&experiments_in_set.biosample.biosource_summary=HFFc6+%28Tier+1%29&experiments_in_set.experiment_type.display_title=DNA+SPRITE&experiments_in_set.experiment_type.display_title=PLAC-seq.

GAM H1-hESC was also downloaded from 4DN Data Portal and can be found here: https://data.4dnucleome.org/search/?q=GAM+H1-hESC&type=Item . No additional processing was applied to GAM data.

Preferential Interactions

Bigwig or bedgraph files for LMNB1 DamID, TSA-Seq and Repli-Seq were downloaded from DCIC Data Portal. The link for those files can be found here : https://data.4dnucleome.org/browse/?dataset_label=E%2FL+repliseq+on+H1-hESC+cells+%282017-08-17%29&dataset_label=E%2FL+repliseq+on+HFFc6+cells+%282017-08-17%29&dataset_label=E%2FL+repliseq+on+HFFc6+cells+-+Gold+Standard++%282018-02-06%29&dataset_label=TSA-seq+MKI67IP+in+H1+cells&dataset_label=TSA-seq+MKI67IP+in+HFFc6+cells&dataset_label=TSA-seq+POL1RE+in+H1+cells&dataset_label=TSA-seq+POL1RE+in+HFFc6+cells&dataset_label=TSA-seq+v2+SON+in+H1&dataset_label=TSA-seq+v2+SON+in+HFFc6&dataset_label=DamID-seq+on+H1-hESC+cells+%282017-12-14%29&dataset_label=DamID-seq+on+HFFc6+cells+%282017-12-14%29&dataset_label=ChIA-PET+in+H1-hESC&dataset_label=ChIA-PET+in+HFFc6&experiments_in_set.biosample.biosource.organism.name=human&experiments_in_set.experiment_categorizer.combined=Target%3A+LMNB1+protein&experiments_in_set.experiment_categorizer.combined=Target%3A+CTCF+protein&experiments_in_set.experiment_categorizer.combined=Target%3A+NIFK+protein&experiments_in_set.experiment_categorizer.combined=Target%3A+POLR1E+protein&experiments_in_set.experiment_categorizer.combined=Target%3A+SON+protein&experiments_in_set.experiment_categorizer.combined=Fraction%3A+early+fraction+of+2+fractions&experiments_in_set.experiment_categorizer.combined=Fraction%3A+late+fraction+of+2+fractions&experiments_in_set.experiment_categorizer.combined=Target%3A+RNA+Pol+II&experiments_in_set.experiment_categorizer.combined=No+value&experimentset_type=replicate&type=ExperimentSetReplicate

2. **Methods for relating chromatin loops to gene expression.**

Datasets

Description	Cell Type	Data Source/Download Link

Loop predictions combined from multiple 4DN experimental assays	H1-ESC<br>HFFc6	https://www.jianguoyun.com/p/DQkAPQkQh9qdDBik0cYFIAA<br>https://www.jianguoyun.com/p/Dd-fjT8Qh9qdDBim0cYFIAA
ATAC-Seq peaks	H1-ESC<br>HFFc6	4DN (4DNFI247OOFU)<br>4DN (4DNFIWQJFZHS)
House-keeping genes in human	-	HRT Atlas v1.0 (https://housekeeping.unicamp.br/)
H3K27ac ChIP-Seq signals	H1-ESC<br>HFFc6	ENCODE (ENCFF986PCY)<br>4DN (4DNFINRI6WOL)
ATAC-Seq signals	H1-ESC<br>HFFc6	4DN (4DNFICPNO4M5)<br>4DN (4DNFIZ9191QU)
CTCF ChIP-Seq signals	H1-ESC<br>HFFc6	ENCODE (ENCFF332TNJ)<br>ENCODE (ENCFF406SZM)
LMNB1 DamID-Seq signals	H1-ESC<br>HFFc6	4DN (4DNFI6BH48Y3)<br>4DN (4DNFI7724Y7Q)
RNA-Seq for 116 tissues / cell types	ovary	ENCODE (ENCFF095OFV, ENCFF940WXY, ENCFF857MSA)
	right renal cortex interstitium	ENCODE (ENCFF320UVF, ENCFF821JAE, ENCFF981FYW)
	hindlimb muscle	ENCODE (ENCFF680ZPA)
	heart right ventricle	ENCODE (ENCFF823DWN, ENCFF102BTQ)
	A549	ENCODE (ENCFF627QMV, ENCFF369ZNM)
	chorionic villus	ENCODE (ENCFF274JIK,ENCFF101AAR, ENCFF529CAT,E NCFF834AEP)
	Panc1	ENCODE (ENCFF890DEQ, ENCFF248YCR)
	luminal epithelial cell of mammary gland	ENCODE (ENCFF047QOH)
	SK-N-SH	ENCODE (ENCFF389TFR, ENCFF161JEA, ENCFF390KQP, ENCFF067ZMG)
	natural killer cell	ENCODE (ENCFF036GDL)

testis	ENCODE (ENCFF850LMK, ENCFF845QSA)
RWPE-1	ENCODE (SRR8446409, SRR8446410, SRR8446411)
aorta	ENCODE (ENCFF277LBD, ENCFF914RDG)
germinal matrix	ENCODE (ENCFF951YSP)
brain	ENCODE (ENCFF784ZTQ)
right lung	ENCODE (ENCFF035KGA, ENCFF535JUK, ENCFF015DMG, ENCFF083YGV, ENCFF487DRK)
endodermal cell	ENCODE (ENCFF563QGW, ENCFF237ZQX)
lung	ENCODE (ENCFF947WLV, ENCFF051UVH, ENCFF014OUE)
liver	ENCODE (ENCFF239EUU, ENCFF908GIP, ENCFF592KZK, ENCFF203UGC)
myoepithelial cell of mammary gland	ENCODE (ENCFF674EKN)
GM23248	ENCODE (ENCFF341SCS, ENCFF775DYT)
endocrine pancreas	ENCODE (ENCFF174RSS, ENCFF982TBJ)
foreskin melanocyte	ENCODE (ENCFF441UUO, ENCFF724NAG)
adrenal gland	ENCODE (ENCFF217TKV, ENCFF802ADF, ENCFF555RGZ, ENCFF467PRR, ENCFF866LBS, ENCFF918DYI, ENCFF739OIE, ENCFF908UKE)
neural cell	ENCODE (ENCFF813LWT, ENCFF081JBX)
fibroblast of lung	ENCODE (ENCFF227FMH, ENCFF983VCS)

MCF10A	ENCODE (SRR5364109, SRR5364108, SRR5364107, SRR5364106)
U-87 MG	ENCODE (ENCFF164HCK, ENCFF334XLV)
peripheral blood mononuclear cell	ENCODE (ENCFF475DKC, ENCFF443WJD)
spleen	ENCODE (ENCFF597SJD, ENCFF693OQP, ENCFF921BKL, ENCFF545TFV, ENCFF809RAX, ENCFF398TQO)
hepatocyte	ENCODE (ENCFF138JDF, ENCFF797ZIB)
neural stem progenitor cell	ENCODE (ENCFF789VZB, ENCFF183XSM)
foreskin fibroblast	ENCODE (ENCFF219FYH, ENCFF964QLH)
MCF7	ENCODE (ENCFF009GDJ, ENCFF885LEQ)
BJ	ENCODE (ENCFF800TGS, ENCFF839MWS)
fibroblast of breast	ENCODE (ENCFF355QNL, ENCFF281CHW)
smooth muscle cell	ENCODE (ENCFF003QAY, ENCFF852RUU)
placenta	ENCODE (ENCFF435PHN)
renal cortex interstitium	ENCODE (ENCFF656GXW, ENCFF885EHC, ENCFF067NHZ)
UCSF-4	ENCODE (ENCFF219WTP)
heart left ventricle	ENCODE (ENCFF860DPP, ENCFF998SEL)
NCI-H460	ENCODE (ENCFF322HJX, ENCFF011JTT, ENCFF522SUJ)
cardiac muscle cell	ENCODE (ENCFF761ACO, ENCFF509IXK)
HEK293	ENCODE (SRR5137672, SRR5137671, SRR5137670)

mesodermal cell	ENCODE (ENCFF553EAV, ENCFF749RUQ)
adipose tissue	ENCODE (ENCFF878UHQ, ENCFF862LZV, ENCFF732LRY, ENCFF272HOG)
GM12878	ENCODE (ENCFF200USH, ENCFF905XDJ, ENCFF644DIQ, ENCFF456WWG, ENCFF876LUX, ENCFF886FDY, ENCFF121ZOP, ENCFF599JTV, ENCFF545OJE, ENCFF853VUK, ENCFF392CRO, ENCFF102NLY, ENCFF315WZE, ENCFF477JYI, ENCFF902UYP, ENCFF550OHK)
CD14-positive monocyte	ENCODE (ENCFF397DFK, ENCFF299BIL, ENCFF219ECV)
skeletal muscle myoblast	ENCODE (ENCFF505GUJ, ENCFF354AZS)
skin fibroblast	ENCODE (ENCFF694YJO, ENCFF202DSC, ENCFF458PJZ, ENCFF959PRH)
muscle of leg	ENCODE (ENCFF125HHE, ENCFF068NMI, ENCFF393OUO, ENCFF884IWB, ENCFF316SOJ, ENCFF559DXJ, ENCFF622TLD, ENCFF114CDE, ENCFF398PFM)
layer of hippocampus	ENCODE (ENCFF323NAG)
HT1080	ENCODE (ENCFF091WWB, ENCFF748NPL, ENCFF241KQK, ENCFF284XTA)
HFFc6	4DN (4DNFI5MR6C3G, 4DNFIF3H5ZCH)
ectodermal cell	ENCODE (ENCFF034KRQ, ENCFF419KMW, ENCFF691ZYQ, ENCFF768SPT)

foreskin keratinocyte	ENCODE (ENCFF680BHT, ENCFF994UBN, ENCFF892CWH, ENCFF051VYX)
PFSK-1	ENCODE (ENCFF635ALW, ENCFF568GAV)
left renal cortex interstitium	ENCODE (ENCFF507HAK, ENCFF068QRU, ENCFF869JVY, ENCFF278ZFZ)
SK-N-DZ	ENCODE (ENCFF635RKM, ENCFF594NJL, ENCFF499DSN, ENCFF611SKK)
chorion	ENCODE (ENCFF550CHR, ENCFF364QYB, ENCFF672UJB, ENCFF397ZBW)
kidney	ENCODE (ENCFF525CMT, ENCFF804WTK, ENCFF099FXO, ENCFF229DFM, ENCFF326MQO)
trophoblast	ENCODE (ENCFF768QIJ, ENCFF591XIE, ENCFF270MHZ, ENCFF873XNT)
left kidney	ENCODE (ENCFF593SHI)
B cell	ENCODE (ENCFF770XDU, ENCFF231GYC, ENCFF485EUP)
GM23338	ENCODE (ENCFF305XIS, ENCFF149CBS)
keratinocyte	ENCODE (ENCFF401JWS, ENCFF344FGV, ENCFF065UCN, ENCFF697CPR, ENCFF734GZX, ENCFF345YOV, ENCFF330VCJ, ENCFF165MYR)
thymus	ENCODE (ENCFF487KXD, ENCFF118YIZ, ENCFF380IZG, ENCFF237HIP, ENCFF432EQO, ENCFF123LVM, ENCFF608SHV)

HepG2	ENCODE (ENCFF640ZBJ, ENCFF861GCR, ENCFF534SLQ, ENCFF945LNB, ENCFF197XZL, ENCFF874RXH, ENCFF401KRE, ENCFF004HYK)
left lung	ENCODE (ENCFF066QDJ, ENCFF919EYT, ENCFF934RBH, ENCFF643UJO, ENCFF391BNP, ENCFF406OKZ)
common myeloid progenitor, CD34-positive	ENCODE (ENCFF690QPA)
AG04450	ENCODE (ENCFF025DRM, ENCFF350BOF)
stomach	ENCODE (ENCFF874WKO, ENCFF031EAX, ENCFF881ZXZ, ENCFF953SNL, ENCFF850QFV, ENCFF815DCS, ENCFF082DAH, ENCFF775TWS, ENCFF648ZHB, ENCFF050VJS)
pancreas	ENCODE (ENCFF625HJC, ENCFF390JAT, ENCFF971GFG)
renal pelvis	ENCODE (ENCFF524ZGN, ENCFF237VRQ, ENCFF013EVX)
T-cell	ENCODE (ENCFF158VJT)
muscle of trunk	ENCODE (ENCFF073LSZ, ENCFF419PGC)
esophagus	ENCODE (ENCFF993JBC, ENCFF566SLH)
293T	ENCODE (SRR12137695, SRR12137698, SRR12137696, SRR12137697)
sigmoid colon	ENCODE (ENCFF474EOZ, ENCFF904HJO, ENCFF362GHJ, ENCFF395HHA)
spinal cord	ENCODE (ENCFF144IMD, ENCFF400RFD, ENCFF340FSS)
forelimb muscle	ENCODE (ENCFF927KFT)

muscle of arm	ENCODE (ENCFF404ZXJ, ENCFF993LHQ, ENCFF350ZWB, ENCFF272NAO, ENCFF726VKQ, ENCFF603FIV, ENCFF905BGI, ENCFF038FYS, ENCFF007LRI, ENCFF513PLX, ENCFF647PIL, ENCFF690YGY)
urinary bladder	ENCODE (ENCFF986LPX)
H1	ENCODE (ENCFF059UBK, ENCFF667AIY, ENCFF741PUY, ENCFF235XMZ, ENCFF113VWX, ENCFF915AUQ, ENCFF653XHG)
mesenchymal stem cell	ENCODE (ENCFF693WRN, ENCFF290OQE)
astrocyte	ENCODE (ENCFF256APB)
psoas muscle	ENCODE (ENCFF543WCB, ENCFF630ZKI, ENCFF489IIG)
NB4	ENCODE (SRR6006856, SRR6006857, SRR6006858)
fibroblast of skin of abdomen	ENCODE (ENCFF327BZW, ENCFF063EGA)
Jurkat clone E61	ENCODE (ENCFF489SJY, ENCFF558JTV)
IMR90	ENCODE (ENCFF244VME, ENCFF118OFK)
mammary epithelial cell	ENCODE (ENCFF380GBC, ENCFF370XTW)
SK-MEL-5	ENCODE (ENCFF620LZN, ENCFF845RTT, ENCFF070UJX, ENCFF448BML)
mesendoderm	ENCODE (ENCFF466QUZ, ENCFF044YLS)
HUVEC	ENCODE (ENCFF917CHS, ENCFF804WUK, ENCFF650CVW, ENCFF238WEU)

small intestine	ENCODE (ENCFF411RSF, ENCFF769FAA, ENCFF309DZJ, ENCFF845OKW, ENCFF647PTK, ENCFF686JQP ,ENCFF537WNL, ENCFF080HMB, ENCFF256ELA, ENCFF970SOK)
heart	ENCODE (ENCFF227RBV, ENCFF089NNQ, ENCFF496OSH)
muscle of back	ENCODE (ENCFF706VUD, ENCFF382FLL, ENCFF313PHN, ENCFF302HTI, ENCFF282SUS, ENCFF424LYQ, ENCFF115ORA, ENCFF195KMJ, ENCFF185TFE, ENCFF860PYR)
neurosphere	ENCODE (ENCFF153QRP, ENCFF707AMK, ENCFF993ZTH)
K562	ENCODE (ENCFF490IGF, ENCFF022QGS, ENCFF768TKT, ENCFF172GIN, ENCFF026BMH, ENCFF868MFR, ENCFF937GNL, ENCFF047WAI, ENCFF427EWZ, ENCFF342LXD, ENCFF472EUD, ENCFF185UMS, ENCFF461HPX, ENCFF156DDL)
CD8-positive, alpha-beta T cell	ENCODE (ENCFF372OMD, ENCFF088DIY)
cerebellum	ENCODE (ENCFF777IQQ)
mammary stem cell	ENCODE (ENCFF692TAL)
amnion	ENCODE (ENCFF144PZJ, ENCFF443KUS, ENCFF416MQP)
placental basal plate	ENCODE (ENCFF345ADJ, ENCFF457JWP, ENCFF434YJO, ENCFF026SBP)

	HeLa-S3	ENCODE (ENCFF846THO, ENCFF796REI, ENCFF010RHX, ENCFF206NFZ, ENCFF344HRY, ENCFF514TNR, ENCFF922LIV, ENCFF101GPJ)
	BE2C	ENCODE (ENCFF396XRF, ENCFF238HUZ)
	HUES64	ENCODE (ENCFF339WEH, ENCFF682DUY)
	left renal pelvis	ENCODE (ENCFF126KII, ENCFF602NKQ, ENCFF720JRI, ENCFF774PTF)
	mole	ENCODE (ENCFF726KPQ)
	right renal pelvis	ENCODE (ENCFF033EGX, ENCFF887YTL, ENCFF494FTI, ENCFF896OFQ)
	trophoblast cell	ENCODE (ENCFF342LYI, ENCFF760HDK)
	Purkinje cell	ENCODE (ENCFF683XBG, ENCFF890PPZ)
	CD4-positive, alpha-beta T cell	ENCODE (ENCFF760LWW, ENCFF794NBU)
	large intestine	ENCODE (ENCFF615SUS, ENCFF688MYF, ENCFF554XMC, ENCFF359YQU, ENCFF972VRS, ENCFF458LNT, ENCFF858BYE)
	right cardiac atrium	ENCODE (ENCFF635SOC)
CTCF ChIA-PET for 32 cell lines or primary cells	CD4-positive, alpha-beta T cell	ENCODE (ENCSR345UIQ)
	activated CD8-positive, alpha-beta T cell (treated with anti-CD3 and anti-CD28 coated beads for 36 hours)	ENCODE (ENCSR408MRB)
	IMR-90	ENCODE (ENCSR076TTY)
	OCI-LY7	ENCODE (ENCSR401JWQ)
	A673	ENCODE (ENCSR549TMF)
	CD4-positive, alpha-beta memory T cell	ENCODE (ENCSR106INW)

CD8-positive, alpha-beta T cell	ENCODE (ENCSR180GEY)
HUVEC	ENCODE (ENCSR404FPI)
activated CD8-positive, alpha-beta memory T cell (treated with anti-CD3 and anti-CD28 coated beads for 36 hours)	ENCODE (ENCSR697ZHG)
activated CD8-positive, alpha-beta memory T cell (treated with 10 ng/mL Interleukin-2 for 5 days, anti-CD3 and anti-CD28 coated beads for 7 days)	ENCODE (ENCSR378IUJ)
activated CD8-positive, alpha-beta T cell (treated with 10 ng/mL Interleukin-2 for 5 days, anti-CD3 and anti-CD28 coated beads for 7 days)	ENCODE (ENCSR531UMR)
naive thymus-derived CD4-positive, alpha-beta T cell	ENCODE (ENCSR120LMS)
K562	ENCODE (ENCSR597AKG)
activated CD4-positive, alpha-beta memory T cell (treated with 10 ng/mL Interleukin-2 for 5 days, anti-CD3 and anti-CD28 coated beads for 7 days)	ENCODE (ENCSR291RCO)
activated CD4-positive, alpha-beta T cell (treated with 10 ng/mL Interleukin-2 for 5 days, anti-CD3 and anti-CD28 coated beads for 7 days)	ENCODE (ENCSR962FFY)
activated T-cell (treated with 50 U/mL Interleukin-2 for 72 hours, anti-CD3 and anti-CD28 coated beads for 72 hours)	ENCODE (ENCSR411UEL, ENCSR038GON)
CD8-positive, alpha-beta memory T cell	ENCODE (ENCSR187PXW)

	T-cell	ENCODE (ENCSR545NUL, ENCSR592BWZ)
	Caco-2	ENCODE (ENCSR185PEE)
	WTC11	ENCODE (ENCSR016VPZ)
	A549	ENCODE (ENCSR911ZMB)
	GM12878	ENCODE (ENCSR184YZV)
	activated CD4-positive, alpha-beta memory T cell (treated with anti-CD3 and anti-CD28 coated beads for 36 hours)	ENCODE (ENCSR154MAJ)
	MCF10A	ENCODE (ENCSR403ZYJ)
	MCF-7	ENCODE (ENCSR200VHL)
	activated B cell (treated with 0.5 μM CpG ODN for 24 hours)	ENCODE (ENCSR494NNF)
	activated naive CD4-positive, alpha-beta T cell (treated with anti-CD3 and anti-CD28 coated beads for 36 hours)	ENCODE (ENCSR731OFF)
	HCT116	ENCODE (ENCSR278IZK)
	activated CD4-positive, alpha-beta T cell (treated with anti-CD3 and anti-CD28 coated beads for 36 hours)	ENCODE (ENCSR093CTT)
	HepG2	ENCODE (ENCSR411IVB)
	Panc1	ENCODE (ENCSR145PYF)
	B-cell	ENCODE (ENCSR536ZNI)
RNAPII ChIA-PET for 32 cell lines or primary cells	CD4-positive, alpha-beta T cell	ENCODE (ENCSR448ZLA)
	activated CD8-positive, alpha-beta T cell (treated with anti-CD3 and anti-CD28 coated beads for 36 hours)	ENCODE (ENCSR982KEM)
	IMR-90	ENCODE (ENCSR966RPQ)
	OCI-LY7	ENCODE (ENCSR882BUM)
	A673	ENCODE (ENCSR623KNI)
	CD4-positive, alpha-beta memory T cell	ENCODE (ENCSR569TBN)

CD8-positive, alpha-beta T cell	ENCODE (ENCSR185VQH)
HUVEC	ENCODE (ENCSR080OMN)
activated CD8-positive, alpha-beta memory T cell (treated with anti-CD3 and anti-CD28 coated beads for 36 hours)	ENCODE (ENCSR041UPG)
activated CD8-positive, alpha-beta memory T cell (treated with 10 ng/mL Interleukin-2 for 5 days, anti-CD3 and anti-CD28 coated beads for 7 days)	ENCODE (ENCSR114KEO)
activated CD8-positive, alpha-beta T cell (treated with 10 ng/mL Interleukin-2 for 5 days, anti-CD3 and anti-CD28 coated beads for 7 days)	ENCODE (ENCSR217TFN)
naive thymus-derived CD4-positive, alpha-beta T cell	ENCODE (ENCSR763OCG)
K562	ENCODE (ENCSR880DSH)
activated CD4-positive, alpha-beta memory T cell (treated with 10 ng/mL Interleukin-2 for 5 days, anti-CD3 and anti-CD28 coated beads for 7 days)	ENCODE (ENCSR159PXF)
activated CD4-positive, alpha-beta T cell (treated with 10 ng/mL Interleukin-2 for 5 days, anti-CD3 and anti-CD28 coated beads for 7 days)	ENCODE (ENCSR733XLQ)
activated T-cell (treated with 50 U/mL Interleukin-2 for 72 hours, anti-CD3 and anti-CD28 coated beads for 72 hours)	ENCODE (ENCSR165FXG, ENCSR891IMI, ENCSR538SBO)
CD8-positive, alpha-beta memory T cell	ENCODE (ENCSR149TQU)

	T-cell	ENCODE (ENCSR722NQM, ENCSR743YTL)
	Caco-2	ENCODE (ENCSR713NCY)
	WTC11	ENCODE (ENCSR972JTN)
	A549	ENCODE (ENCSR138NSW)
	GM12878	ENCODE (ENCSR905HWW)
	activated CD4-positive, alpha-beta memory T cell (treated with anti-CD3 and anti-CD28 coated beads for 36 hours)	ENCODE (ENCSR877ZRR)
	MCF10A	ENCODE (ENCSR499JGQ)
	MCF-7	ENCODE (ENCSR059HDE)
	activated B cell (treated with 0.5 μM CpG ODN for 24 hours)	ENCODE (ENCSR096ZPB)
	activated naive CD4-positive, alpha-beta T cell (treated with anti-CD3 and anti-CD28 coated beads for 36 hours)	ENCODE (ENCSR402NAQ)
	HCT116	ENCODE (ENCSR035PVZ)
	activated CD4-positive, alpha-beta T cell (treated with anti-CD3 and anti-CD28 coated beads for 36 hours)	ENCODE (ENCSR314TNQ)
	HepG2	ENCODE (ENCSR789ZIJ, ENCSR857MYZ)
	Panc1	ENCODE (ENCSR447IUA)
	B-cell	ENCODE (ENCSR172WWJ)
DNase-Seq peaks for 32 cell lines or primary cells	CD4-positive, alpha-beta T cell	ENCODE (ENCFF138ZRF, ENCFF396CHT, ENCFF348SDZ, ENCFF147ZBC)
	activated CD8-positive, alpha-beta T cell (treated with anti-CD3 and anti-CD28 coated beads for 36 hours)	ENCODE (ENCFF834QBM)
	IMR-90	ENCODE (ENCFF800DVI, ENCFF525PWS)

OCI-LY7	ENCODE (ENCFF196DIZ, ENCFF903CVK)
A673	ENCODE (ENCFF607YBN, ENCFF355NVJ, ENCFF606OTK)
CD4-positive, alpha-beta memory T cell	ENCODE (ENCFF157MKK, ENCFF967NLQ, ENCFF386UFI, ENCFF010BIN)
CD8-positive, alpha-beta T cell	ENCODE (ENCFF070WZM, ENCFF792FLJ, ENCFF326MXR, ENCFF017FZE)
HUVEC	ENCODE (ENCFF833UNB, ENCFF136MYL, ENCFF406AWN)
activated CD8-positive, alpha-beta memory T cell (treated with anti-CD3 and anti-CD28 coated beads for 36 hours)	ENCODE (ENCFF401WTQ, ENCFF848EBC, ENCFF713XCT, ENCFF810ZJY)
activated CD8-positive, alpha-beta memory T cell (treated with 10 ng/mL Interleukin-2 for 5 days, anti-CD3 and anti-CD28 coated beads for 7 days)	ENCODE (ENCFF792PYU)
activated CD8-positive, alpha-beta T cell (treated with 10 ng/mL Interleukin-2 for 5 days, anti-CD3 and anti-CD28 coated beads for 7 days)	ENCODE (ENCFF596UGI)
naive thymus-derived CD4-positive, alpha-beta T cell	ENCODE (ENCFF885PJD, ENCFF937FLI, ENCFF901UGO, ENCFF951XVJ)
K562	ENCODE (ENCFF274YGF, ENCFF264UFX, ENCFF185XRG, ENCFF807ICZ)
activated CD4-positive, alpha-beta memory T cell (treated with 10 ng/mL Interleukin-2 for 5 days, anti-	ENCODE (ENCFF552TYH)

CD3 and anti-CD28 coated beads for 7 days)	
activated CD4-positive, alpha-beta T cell (treated with 10 ng/mL Interleukin-2 for 5 days, anti-CD3 and anti-CD28 coated beads for 7 days)	ENCODE (ENCFF765OGD)
activated T-cell (treated with 50 U/mL Interleukin-2 for 72 hours, anti-CD3 and anti-CD28 coated beads for 72 hours)	ENCODE (ENCFF725EBY, ENCFF168GAN)
CD8-positive, alpha-beta memory T cell	ENCODE (ENCFF931AKV, ENCFF375PHK, ENCFF041GGN, ENCFF224KBE)
T-cell	ENCODE (ENCFF873FYV, ENCFF729DOV, ENCFF933QZD, ENCFF839QLN)
Caco-2	ENCODE (ENCFF810WJX, ENCFF637OJO, ENCFF948AMM, ENCFF579UXQ)
WTC11	ENCODE (ENCFF668BJR, ENCFF854DSG)
A549	ENCODE (ENCFF128ZVL, ENCFF410KIB, ENCFF302JWZ)
GM12878	ENCODE (ENCFF338SAU, ENCFF759OLD)
activated CD4-positive, alpha-beta memory T cell (treated with anti-CD3 and anti-CD28 coated beads for 36 hours)	ENCODE (ENCFF067SGU)
MCF10A	ENCODE (ENCFF667FTX)
MCF-7	ENCODE (ENCFF107HQA, ENCFF886OJN, ENCFF835KCG, ENCFF536CIK)

activated B cell (treated with 0.5 μM CpG ODN for 24 hours)	ENCODE (ENCFF489NNB)
activated naive CD4-positive, alpha-beta T cell (treated with anti-CD3 and anti-CD28 coated beads for 36 hours)	ENCODE (ENCFF261QWU)
HCT116	ENCODE (ENCFF356GTJ, ENCFF240LRP)
activated CD4-positive, alpha-beta T cell (treated with anti-CD3 and anti-CD28 coated beads for 36 hours)	ENCODE (ENCFF107XDE)
HepG2	ENCODE (ENCFF748QCZ, ENCFF897NME, ENCFF453AEP)
Panc1	ENCODE (ENCFF415BYA, ENCFF842AOT)
B-cell	ENCODE (ENCFF262LZH, ENCFF245RKH, ENCFF526MQV, ENCFF248ACA)

3. Method for SPIN states identification and analysis

Data availability

Cell Type	Experiment Type	Data Type	Data Source/Download Link	Note1
H1-ESC	SON TSA-seq	Signal (BigWig)	4DN (4DNESUTK6QWG, 4DNESC3D6NGQ)	
	LaminB TSA-seq	Signal (BigWig)	4DN (4DNESHZ8WKRX, 4DNESGGXKI1H)	
	Nucleous TSA-seq	Signal (BigWig)	4DN (4DNESO6HFSAD, 4DNES6PANOF4)	
	LaminB DamID	Signal (BigWig)	4DN (4DNESOFQR5FS, 4DNESXKBPZKQ)	
	Hi-C	Contact matrix	4DN (4DNESX75DD7R, 4DNES2M5JIGV)	

	H2AFZ ChIP-Seq	Signal (BigWig)	ENCODE (ENCFF758YFI)	
	H3K27ac ChIP-Seq	Signal (BigWig)	ENCODE (ENCFF771GNB)	
	H3K27me3 ChIP-Seq	Signal (BigWig)	ENCODE (ENCFF277UCT)	
	H3K36me3 ChIP-Seq	Signal (BigWig)	ENCODE (ENCFF687LJF)	
	H3K4me1 ChIP-Seq	Signal (BigWig)	ENCODE (ENCFF335ZGP)	
	H3K4me2 ChIP-Seq	Signal (BigWig)	ENCODE (ENCFF501AUN)	
	H3K4me3 ChIP-Seq	Signal (BigWig)	ENCODE (ENCFF698DKQ)	
	H3K79me2 ChIP-Seq	Signal (BigWig)	ENCODE (ENCFF640WRD)	
	H3K9ac ChIP-Seq	Signal (BigWig)	ENCODE (ENCFF890MIB)	
	H3K9me3 ChIP-Seq	Signal (BigWig)	ENCODE (ENCFF358AWN)	
	H4K20me1 ChIP-Seq	Signal (BigWig)	ENCODE (ENCFF453OCL)	
	16-fraction Repli-seq G1	Alignment (Bam)	4DN (4DNES2W2DXM6)	The smoothed and normalized RT score can be downloaded from GSE137764
	16-fraction Repli-seq S1	Alignment (Bam)	4DN (4DNES8GLHOD6)	
	16-fraction Repli-seq S2	Alignment (Bam)	4DN (4DNESWWMQ1IC)	
	16-fraction Repli-seq S3	Alignment (Bam)	4DN (4DNES2LV4C4Q)	
	16-fraction Repli-seq S4	Alignment (Bam)	4DN (4DNES2RXY8IB)	
	16-fraction Repli-seq S5	Alignment (Bam)	4DN (4DNESVX7IH76)	
	16-fraction Repli-seq S6	Alignment (Bam)	4DN (4DNESLOM1MB7)	
	16-fraction Repli-seq S7	Alignment (Bam)	4DN (4DNESR9FVS6K)	
	16-fraction Repli-seq S8	Alignment (Bam)	4DN (4DNESFMEOEHW)	
	16-fraction Repli-seq S9	Alignment (Bam)	4DN (4DNESBZIJJ1E)	

	16-fraction Repli-seq S10	Alignment (Bam)	4DN (4DNESDZOL2VU)	
	16-fraction Repli-seq S11	Alignment (Bam)	4DN (4DNESJJKOIH4)	
	16-fraction Repli-seq S12	Alignment (Bam)	4DN (4DNES4I9OXS7)	
	16-fraction Repli-seq S13	Alignment (Bam)	4DN (4DNES6G2SSN2)	
	16-fraction Repli-seq S14	Alignment (Bam)	4DN (4DNESGY7RRTV)	
	16-fraction Repli-seq S15	Alignment (Bam)	4DN (4DNESYIVVMOC)	
	16-fraction Repli-seq S16	Alignment (Bam)	4DN (4DNESIK2HK47)	
	iMARGI	Read pairs	4DN (4DNESOBRUQ12, 4DNES8B3R3P8, 4DNESGRI8A8N, 4DNESNOJ7HY7)	Only interchromosomal repetitive element (RE)-containing chromatin-associated RNAs (caRNAs) read pairs are used
HFFc6	SON TSA-seq	Signal (BigWig)	4DN (4DNESB5I8TGR, 4DNES85R9TIB)	
	LaminB TSA-seq	Signal (BigWig)	4DN (4DNES16C6XVY, 4DNESMF4T7QQ)	
	Nucleous TSA-seq	Signal (BigWig)	4DN (4DNESGAR9ZBW, 4DNES2RN8ZJ1)	
	LaminB DamID	Signal (BigWig)	4DN (4DNESOOCOBBA, 4DNESXZ4FW4T)	
	Hi-C	Contact matrix	4DN (4DNESNMAAN97, 4DNES2R6PUEK)	
	Imputed H2AFZ ChIP-Seq	Signal (BigWig)	4DN (4DNFIB79WDUY)	
	Imputed H3K27ac ChIP-Seq	Signal (BigWig)	4DN (4DNFIBNZ478I)	
	Imputed H3K27me3 ChIP-Seq	Signal (BigWig)	4DN (4DNFIMJHO898)	
	Imputed H3K36me3 ChIP-Seq	Signal (BigWig)	4DN (4DNFIDU8WT76)	
	Imputed H3K4me1 ChIP-Seq	Signal (BigWig)	4DN (4DNFIBC1VSF6)	

	Imputed H3K4me2 ChIP-Seq	Signal (BigWig)	4DN (4DNFIFB7SHA1)	
	Imputed H3K4me3 ChIP-Seq	Signal (BigWig)	4DN (4DNFIKRPF9QP)	
	Imputed H3K79me2 ChIP-Seq	Signal (BigWig)	4DN (4DNFIBS37Z1K)	
	Imputed H3K9ac ChIP-Seq	Signal (BigWig)	4DN (4DNFINBZ9NNY)	
	Imputed H3K9me3 ChIP-Seq	Signal (BigWig)	4DN (4DNFILJFE7WW)	
	Imputed H4K20me1 ChIP-Seq	Signal (BigWig)	4DN (4DNFI13RAB7H)	
	CUT&RUN CTCF	Raw (fastq)	4DN (4DNES1RQBHPK)	
	CUT&RUN H2A.Z	Raw (fastq)	4DN (4DNESHPUFWTR)	
	CUT&RUN H3K27ac	Raw (fastq)	4DN (4DNESIMWCLF8)	
	CUT&RUN H3K27me3	Raw (fastq)	4DN (4DNES8TY5P5P)	
	CUT&RUN H3K4me1	Raw (fastq)	4DN (4DNESRFWR5SV)	
	CUT&RUN H3K4me2	Raw (fastq)	4DN (4DNESEQW5QAI)	
	CUT&RUN H3K4me3	Raw (fastq)	4DN (4DNESPE6J9FU)	
	iMARGI	Read pairs	4DN (4DNES9Y1GHK4)	Only interchromosomal repetitive element (RE)-containing chromatin-associated RNAs (caRNAs) read pairs are used

The iMARGI datasets used for this paper are as follows:

Cell line	DCIC accession

H1	4DNESNOJ7HY7
HFFc6	4DNES9Y1GHK4
K562	4DNESIKCVASO

The bigwig tracks of iMARGI's RNA reads are as follows:

Cell line	Treatmen t	Strand	DCIC accession
H1	Control	Plus	4DNFIOYVWEYZ
H1	Control	Minus	4DNFI2LXIREI

4. **Methods for Integrated Genome Structure Modeling**

Datasets

The 4DN accession codes to the input data used in simulating and analyzing the genome structures are given in the table below.

	**Hi-ESC**	**HFFc6**
**Ensemble Hi-C**	4DNESX75DD7R {Akgol Oksuz, 2021 #1887}	4DNESNMAAN97 {Akgol Oksuz, 2021 #1887}
**Lamina DamID**	4DNESXKBPZKQ	4DNESXZ4FW4T
**SPRITE**	4DNESASBN1JK {Bhat, 2023 #1958}	4DNESJYGTI8S {Bhat, 2023 #1958}
**TSA-seq**	4DNFI625PP2A {Zhang, 2021 #1892}	4DNFI6FTPH5V {Zhang, 2021 #1892}
**RNA-seq**	4DNES3IOYG74	4DNESFH3EHTU

	GSE75748 {Chu, 2016 #1959}	GSE75748 {Chu, 2016 #1959}
**Histone ChIP-seq**	ENCFF986PCY {Roadmap Epigenomics Consortium, 2015 #1286}<br>ENCFF088MXE {Roadmap Epigenomics Consortium, 2015 #1286}<br>ENCFF084JKQ {Roadmap Epigenomics Consortium, 2015 #1286}<br>ENCFF183MHJ {Roadmap Epigenomics Consortium, 2015 #1286}<br>ENCFF860NVB {Roadmap Epigenomics Consortium, 2015 #1286}<br>ENCFF401PZS {Roadmap Epigenomics Consortium, 2015 #1286}<br>ENCFF156JZY {Roadmap Epigenomics Consortium, 2015 #1286}<br>ENCFF065VIF {Roadmap Epigenomics Consortium, 2015 #1286}<br>ENCFF445UVT {Roadmap Epigenomics Consortium, 2015 #1286}<br>ENCFF488THD {Roadmap Epigenomics Consortium, 2015 #1286}<br>ENCFF780FNS {Roadmap Epigenomics Consortium, 2015 #1286}	ENCFF426TLD {ENCODE-Project-Consortium, 2012 #1015}<br>ENCFF792IOR {ENCODE-Project-Consortium, 2012 #1015}<br>ENCFF994SSG {ENCODE-Project-Consortium, 2012 #1015}<br>ENCFF690KUY {ENCODE-Project-Consortium, 2012 #1015}<br>ENCFF995LLA {ENCODE-Project-Consortium, 2012 #1015}<br>ENCFF070SWD {ENCODE-Project-Consortium, 2012 #1015}

5. Methods for single cell 3D genome analysis

scHi-C data

ScHi-C data can be found here:

For clone 6:

https://data.4dnucleome.org/experiment-set-replicates/4DNESJQ4RXY5/

and for clone 28:

https://data.4dnucleome.org/experiment-set-replicates/4DNESF829JOW/

Data accessibility

Hi-C

- h1ESC Hi-C 2.5 source: https://data.4dnucleome.org/files-processed/4DNFI82R42AD/
- h1ESC dot domains: https://data.4dnucleome.org/files-processed/4DNFIW5EIIO2/
- h1ESC dotless domains: https://data.4dnucleome.org/files-processed/4DNFIU7GTTMW/
- h1ESC dot boundaries: https://data.4dnucleome.org/files-processed/4DNFIWNJ5RR7/
- h1ESC dotless boundaries: https://data.4dnucleome.org/files-processed/4DNFIT6QE9YU/
- h1ESC loops: https://data.4dnucleome.org/files-processed/4DNFIEEF14ST/
- h1ESC TADs/subTADs: https://data.4dnucleome.org/files-processed/4DNFIR94OF6S/

16 fraction Repli-seq

- h1ESC raw fastq: https://data.4dnucleome.org/experiment-sets/4DNESXRBILXJ/
- h1ESC read depth scaled normalized array for IZ calls & visualization: https://data.4dnucleome.org/files-processed/4DNFI3N8GHKR/
- h1ESC Early, Early-mid, Late IZs on read depth normalized: https://data.4dnucleome.org/files-processed/4DNFIRF7WZ3H

RNA

- h1ESC RNA-seq source: https://www.encodeproject.org/experiments/ENCSR537BCG/
- h1ESC RNA-seq processed file: https://www.encodeproject.org/files/ENCFF584VXW/ (plus strand signal of unique reads) https://www.encodeproject.org/files/ENCFF307LLA/ (minus strand signal of unique reads)
- h1ESC raw files: http://sysbiocomp.ucsd.edu/public/wenxingzhao/H1_new/05-09-2019_Tri_iMARGI_H1-control.RNA.minus.bw;

http://sysbiocomp.ucsd.edu/public/wenxingzhao/H1_new/05-09-2019_Tri_iMARGI_H1- control.RNA.plus.bw

SPIN states

- h1ESC and HFFc6 SPIN states are in Supplemental Table 1.

h1ESC

- h1ESC A/B Compartments: https://data.4dnucleome.org/files-processed/4DNFIOUDCJRH/

Contact map

- H1ESC: https://data.4dnucleome.org/files-processed/4DNFIIMZB6Y9/

ATAC-seq

- HCT116: https://www.encodeproject.org/files/ENCFF624HRW/
- mESC: https://data.4dnucleome.org/files-processed/4DNFI6HY3NE7/
- H1ESC: https://data.4dnucleome.org/files-processed/4DNFICPNO4M5/

Histones marks

- H1ESC, H2AZ: https://data.4dnucleome.org/experiments-seq/4DNEXHDA1L74/ (reanalyzed)
- H1ESC, H3K27AC: https://data.4dnucleome.org/experiments-seq/4DNEX5EQJ2P2/ (reanalyzed)
- H1ESC, H3K4me3: https://data.4dnucleome.org/experiment-set-replicates/4DNESPE6J9FU
- H1ESC, H3K4me1: https://data.4dnucleome.org/experiments-seq/4DNEXOKGNAEQ/ (reanalyzed)
- HCT116, H2AZ: https://data.4dnucleome.org/files-processed/4DNFIPHO57I2/
- HCT116, H3K27Ac: https://data.4dnucleome.org/files-processed/4DNFIYNC2EO5/
- HCT116, H3K4me3: https://data.4dnucleome.org/files-processed/4DNFIT9DN6JI/
- mESC, H3K27Ac: https://data.4dnucleome.org/files-processed/4DNFIXE23VC7/
- mESC, H3K4me3: https://data.4dnucleome.org/files-processed/4DNFIQYJLKKH/
- mESC H2AZ: https://www.ncbi.nlm.nih.gov/geo/query/acc.cgi?acc=GSM4802378 (reanalyzed)

Gene expression

- HCT116: https://www.encodeproject.org/files/ENCFF766TYC/
- mESC: https://data.4dnucleome.org/files-processed/4DNFI4XVSIFH/

IZs

H1ESC	IZs	4DNFI5OBN63G	https://data.4dnucleome.org/files-processed/4DNFI5OBN63G
HCT116	IZs	4DNFIYO3H24N	https://data.4dnucleome.org/files-processed/4DNFILNNSFMD/
mESC	IZs	4DNFI53E1A38	https://data.4dnucleome.org/files-processed/4DNFI53E1A38/

2-fractions repli-seq

- H1ESC: https://data.4dnucleome.org/experiments-seq/4DNFIWSH27RS ; https://data.4dnucleome.org/experiments-seq/4DNFIQ6F59NT
- HCT116: https://data.4dnucleome.org/files-processed/4DNFIM9S18WO/ ; https://data.4dnucleome.org/files-processed/4DNFIC4VUF86
- mESC: https://data.4dnucleome.org/files-processed/4DNFIPB7M5B6 ; https://data.4dnucleome.org/files-processed/4DNFIRHK7RZF

Insulation score

- H1ESC: https://data.4dnucleome.org/files-processed/4DNFIUK3UVZX
- HCT116: https://data.4dnucleome.org/files-processed/4DNFIGKFF445/
- mESC: https://data.4dnucleome.org/files-processed/4DNFI2LE1KZL/

iMARGI datasets

- h1ESC: https://data.4dnucleome.org/experiment-set-replicates/4DNESNOJ7HY7/
