# Supplementary material for: An integrated view of the structure and function of the human 4D nucleome
Source: Nature. 2025 Dec 17;649(8097):759–76. doi: 10.1038/s41586-025-09890-3 (PMC12804090; doi:10.1038/s41586-025-09890-3)
Supplement: Supplementary file 2 — Reporting Summary [file 41586_2025_9890_MOESM2_ESM.pdf]

nature portfolio

Corresponding author(s): Job Dekker; Xiaotao Wang; Mario Nicodemi; Bing Ren; Sheng Zhong; Jennifer E. Phillips-Cremins; David M. Gilbert; Katherine S. Pollard; Frank Alber; Jian Ma; William S. Noble; Feng Yue

Last updated by author(s): Oct 26, 2024

Reporting Summary

Nature Portfolio wishes to improve the reproducibility of the work that we publish. This form provides structure for consistency and transparency in reporting. For further information on Nature Portfolio policies, see our Editorial Policies and the Editorial Policy Checklist.

Statistics

For all statistical analyses, confirm that the following items are present in the figure legend, table legend, main text, or Methods section.

n/a	Confirmed	
☐	☒	The exact sample size (*n*) for each experimental group/condition, given as a discrete number and unit of measurement
☐	☒	A statement on whether measurements were taken from distinct samples or whether the same sample was measured repeatedly
☐	☒	The statistical test(s) used AND whether they are one- or two-sided<br>*Only common tests should be described solely by name; describe more complex techniques in the Methods section.*
☐	☒	A description of all covariates tested
☐	☒	A description of any assumptions or corrections, such as tests of normality and adjustment for multiple comparisons
☐	☒	A full description of the statistical parameters including central tendency (e.g. means) or other basic estimates (e.g. regression coefficient) AND variation (e.g. standard deviation) or associated estimates of uncertainty (e.g. confidence intervals)
☐	☒	For null hypothesis testing, the test statistic (e.g. *F*, *t*, *r*) with confidence intervals, effect sizes, degrees of freedom and *P* value noted<br>*Give P values as exact values whenever suitable.*
☒	☐	For Bayesian analysis, information on the choice of priors and Markov chain Monte Carlo settings
☒	☐	For hierarchical and complex designs, identification of the appropriate level for tests and full reporting of outcomes
☐	☒	Estimates of effect sizes (e.g. Cohen's *d*, Pearson's *r*), indicating how they were calculated

Our web collection on statistics for biologists contains articles on many of the points above.

Software and code

Policy information about availability of computer code

Data collection

Data from the 4D Nucleome Data Portal were manually downloaded. Data from the ENCODE portal were retrieved using a customized Python script, with the metadata file (metadata.tsv downloaded from ENCODE) as input. We have deposited this script along with the metadata.tsv file on GitHub at the following link https://github.com/XiaoTaoWang/4DN-joint-analysis/tree/main/data-collection.

iMARGI data for H1 or HFF cell lines were downloaded from: https://data.4dnucleome.org/experiment-set-replicates/4DNESNOJ7HY7/#processed-files for H1 and https://data.4dnucleome.org/experiment-set-replicates/4DNES9Y1GHK4/#processed-files for HFF. .iMARGI RNA ends genome wide coverage were downloaded from: https://data.4dnucleome.org/experiment-set-replicates/4DNESNOJ7HY7/#supplementary-files. Genome-wide SPIN states annotation were downloaded from: https://data.4dnucleome.org/joint-analysis#spin-states.

This study analyzed data publicly available through the 4D Nucleome Data Portal (https://data.4dnucleome.org; https://data.4dnucleome.org/joint-analysis) and the ENCODE data portal (https://screen.encodeproject.org/; https://www.encodeproject.org) (See Supplementary note 1). We refer to the Supplementary Materials for a full list of all data accession numbers and public links. This study produced three new datasets that are publicly available: loop calls in H1-hESCs and HFFc6 cells (https://doi.org/10.5281/zenodo.17451616; https://data.4dnucleome.org/files-processed/4DNFIX6VZKOA/; https://data.4dnucleome.org/files-processed/4DNFI32J1C6W/), SPIN state annotations for H1-hESCs and HFFc6 cells (https://data.4dnucleome.org/joint-analysis#spin-states; https://data.4dnucleome.org/files-processed/4DNFITDBR2LR/; https://data.4dnucleome.org/files-processed/4DNFI22FB8HD/), and 3D models and 3D structure feature profiles (https://zenodo.org/records/17459402; https://data.4dnucleome.org/files-processed/4DNFIJ36KR6X/; https://data.4dnucleome.org/files-processed/4DNFILC1YWO6/; https://data.4dnucleome.org/files-processed/4DNFIHF82NAI/; https://data.4dnucleome.org/files-processed/4DNFITOP9BQE/).

Data analysis

Code availability
All code applied in this paper is publicly available through these links:
Methods benchmarking:
https://doi.org/10.5281/zenodo.17475348
GAM analysis
https://doi.org/10.5281/zenodo.17477229
Loop calling, and transcription-loop analysis:
https://doi.org/10.5281/zenodo.17456776
SPIN analysis:
https://doi.org/10.5281/zenodo.17469228.
iMargi analysis:
https://doi.org/10.5281/zenodo.17502239
3D genome structure modeling: Integrative Genome Modeling (IGMv2.0) platform
https://doi.org/10.5281/zenodo.17478448
www.github.com/alberlab/igm
Single cell analysis:
https://doi.org/10.5281/zenodo.17477302
TADs, SPIN, Compartment, and replication analysis:
https://doi.org/10.5281/zenodo.17467184
Replication timing analysis
https://doi.org/10.5281/zenodo.17485344
Predicting Hi-C data from sequence:
https://doi.org/10.5281/zenodo.17469981

FIMO (version 5.5.2) (Grant et al., 2011) and the JASPAR database (2023) (Castro-Mondragon et al., 2022) were used to identify transcription factor binding sites in the hg38 human genome assembly. In -house script was used to identify binding sites overlapping topologically associating domain (TAD) boundaries. The Akita model (https://github.com/calico/basenji/tree/master/manuscripts/akita) (Fudenberg et al. 2020) was used to quantify the effects of sequence changes on genome folding. DeepExplainer (DeepSHAP implementation of DeepLIFT) (version 0.40.0) (Avsec et al., 2021; Shrikumar et al., 2019) was used to visualize the resulting contribution scores.

3D modeling display items: The analyses and most of the figure panels were performed using custom Python scripts (matplotlibv3.5, scikit-learnv1.0, scipyv1.5 and networkxv2.8) together with the publicly available alabtools platform (https://github.com/alberlab/alabtools). Spatial partitions were identified using the MCL algorithm (https://micans.org/mcl/). Images of 3D genome structures were generated using UCSF Chimera1.13.

Chromatin loops were identified using multiple tools depending on the assay type. For Hi-C and Micro-C, loops were called using cooltools (v0.5.1, dots subcommand; https://github.com/open2c/cooltools) and Peakachu (v2.3; https://github.com/tariks/peakachu). For ChIA-PET, loops were identified using ChiaSig (v1.19.44-r2; https://github.com/cheehongsg/ChiaSigScaled) and Peakachu (v2.3). For PLAC-Seq, loops were identified using MAPS (v1.1.0; https://github.com/ijuric/MAPS), ChiaSig (v1.19.44-r2), and Peakachu (v2.3).

Consensus chromatin states were calculated using ChromHMM (v1.23; https://compbio.mit.edu/ChromHMM/), with input ChIP-Seq tracks downloaded from the WashU Epigenome Browser and converted from hg19 to hg38 using CrossMap (v0.5.2; http://crossmap.sourceforge.net/). UMAP projections of chromatin loops were generated using the umap package (v0.5.5; https://github.com/lmcinnes/umap).

The software Higashi (https://github.com/ma-compbio/Higashi) is used for imputing the sparse scHi-C contact maps. The software SPIN (https://github.com/ma-compbio/SPIN) is used for SPIN states inference.
.
For SnapHiC: We used R version 4.2.2, Python version 3.6. SnapHiC version v0.2.3 code is available at: https://github.com/HuMingLab/SnapHiC.

Some Hi-C maps were drawn using ORCA (https://doi.org/10.1038/s41588-022-01065-4)

For manuscripts utilizing custom algorithms or software that are central to the research but not yet described in published literature, software must be made available to editors and reviewers. We strongly encourage code deposition in a community repository (e.g. GitHub). See the Nature Portfolio guidelines for submitting code & software for further information.

Data

Policy information about availability of data

All manuscripts must include a data availability statement. This statement should provide the following information, where applicable:

- Accession codes, unique identifiers, or web links for publicly available datasets
- A description of any restrictions on data availability
- For clinical datasets or third party data, please ensure that the statement adheres to our policy

All data are available at 4DN data portal unless stated otherwise in the manuscript. The supplementary materials includes exhaustive lists of all dataset used in the manuscript, and provides all links. All data is publicly available.

This study analyzed data publicly available through the 4D Nucleome Data Portal (https://data.4dnucleome.org; https://data.4dnucleome.org/joint-analysis) and the ENCODE data portal (https://screen.encodeproject.org/; https://www.encodeproject.org). We refer to the Supplementary Materials for a full list of all data

accession numbers and public links. This study produced three new datasets that are publicly available: loop calls in H1-hESCs and HFFc6 cells (https://doi.org/10.5281/zenodo.17451616), SPIN state annotations for H1-hESCs and HFFc6 cells (https://data.4dnucleome.org/joint-analysis#spin-states; https://data.4dnucleome.org/files-processed/4DNFITDBR2LR/; https://data.4dnucleome.org/files-processed/4DNFI22FB8HD/), and 3D models and 3D structure feature profiles (https://zenodo.org/records/17459402).

For chromatin interaction assays: We collected all the benchmarking and supporting data from the 4D Nucleome Data Portal (https://data.4dnucleome.org/) and the ENCODE data portal (https://www.encodeproject.org/). Detailed information, including the data type, accession code, and download link for each dataset, is provided in the Supplementary Materials.

iMARGI data for H1 or HFF cell lines were downloaded from: https://data.4dnucleome.org/experiment-set-replicates/4DNESNOJ7HY7/#processed-files for H1 and https://data.4dnucleome.org/experiment-set-replicates/4DNES9Y1GHK4/#processed-files for HFF. .iMARGI RNA ends genome wide coverage were downloaded from: https://data.4dnucleome.org/experiment-set-replicates/4DNESNOJ7HY7/#supplementary-files.

WTC-11 scHi-C data is collected from the 4DN Data Portal (https://data.4dnucleome.org/) under accession number 4DNESF829JOW, 4DNESJQ4RXY5. We obtained TSA-seq, Lamin-B DamID, and Hi-C data for H1-hESCs and HFFc6 from the 4DN data portal (http://data.4dnucleom.rog). Data accession IDs are datasets are provided in the supplementary materials. The predicted SPIN states for H1 and HFF are uploaded to 4DN data portal with the accession ID 4DNFI22FB8HD and 4DNFITDBR2LR for H1-hESCs and HFFc6, respectively.

3D models: The genome structure population and genome-wide structural features are available at https://doi.org/10.5281/zenodo.7352276. The accession codes for the experimental data used in our analyses are as follows. Ensemble Hi-C [4DN] H1-ESC: 4DNESX75DD7R, HFFc6: 4DNESNMAAN97 Lamina DamID [4DN] H1-ESC: 4DNESXKBPZKQ, HFFc6: 4DNESXZ4FW4T SPRITE [4DN] H1-ESC: 4DNESASBN1JK, HFFc6: 4DNESJYGTI8S TSA-seq [4DN] H1-ESC: 4DNFI625PP2A, HFFc6: 4DNFI6FTPH5V RNA-seq [4DN, GEO] H1-ESC: 4DNES3IOYG74, GSE75748, HFFc6: 4DNESFH3EHTU, GSE75748 Histone ChIP-seq [ENCODE] H1-ESC: ENCFF986PCY, ENCFF088MXE, ENCFF084JKQ, ENCFF183MHJ, ENCFF860NVB, ENCFF401PZS, ENCFF156JZY, ENCFF065VIF, ENCFF445UVT, ENCFF488THD, ENCFF780FNS, HFFc6: ENCFF426TLD, ENCFF792IOR, ENCFF994SSG, ENCFF690KUY, ENCFF995LLA, ENCFF070SWD

Research involving human participants, their data, or biological material

Policy information about studies with human participants or human data. See also policy information about sex, gender (identity/presentation), and sexual orientation and race, ethnicity and racism.

Reporting on sex and gender	This is not relevant to our study. Our study is based on cell lines.
Reporting on race, ethnicity, or other socially relevant groupings	This is not relevant to our study. Our study is based on cell lines.
Population characteristics	This is not relevant to our study. Our study is based on cell lines.
Recruitment	This is not relevant to our study. Our study is based on cell lines.
Ethics oversight	This is not relevant to our study. Our study is based on cell lines.

Note that full information on the approval of the study protocol must also be provided in the manuscript.

Field-specific reporting

Please select the one below that is the best fit for your research. If you are not sure, read the appropriate sections before making your selection.

☒ Life sciences ☐ Behavioural & social sciences ☐ Ecological, evolutionary & environmental sciences

For a reference copy of the document with all sections, see nature.com/documents/nr-reporting-summary-flat.pdf

Life sciences study design

All studies must disclose on these points even when the disclosure is negative.

Sample size	There are 188 cells in the WTC-11 scHi-C dataset. No sample size calculation was performed. This is the number of cells that passed that passed our internal threshold for sufficient read coverage.<br><br>The number of cells to be used for high-resolution repli--seq was determined so the final sequencing libraries do not have notable PCR duplicates.<br><br>The chromatin structure population contains 1,000 models.
Data exclusions	In general: No data or 3D models were excluded from analysis.<br>For single cell analysis: Ren lab filtered the dataset to get 188 cells so that each cell has sufficient read coverage
Replication	All 3D genome mapping assays analyzed in this study were performed with at least two biological replicates to ensure the reproducibility of the results.

	Each high-resolution repli--seq was done as only one replicate. However, in this multi-fraction assay, each fraction serves as control of other fractions.
Randomization	In general: This is not relevant to our study. Our study does not involve any allocations of samples/organisms/participants.<br><br>In the case of 3D models, this can be relevant: All our genome population calculations (via IGM) start out with fully randomized genome configurations.
Blinding	This not relevant to our study. Our study does not involve any group allocations.

Reporting for specific materials, systems and methods

We require information from authors about some types of materials, experimental systems and methods used in many studies. Here, indicate whether each material, system or method listed is relevant to your study. If you are not sure if a list item applies to your research, read the appropriate section before selecting a response.

Materials & experimental systems

n/a	Involved in the study
☐	☒ Antibodies
☐	☒ Eukaryotic cell lines
☒	☐ Palaeontology and archaeology
☒	☐ Animals and other organisms
☒	☐ Clinical data
☒	☐ Dual use research of concern
☒	☐ Plants

Methods

n/a	Involved in the study
☒	☐ ChIP-seq
☐	☒ Flow cytometry
☒	☐ MRI-based neuroimaging

Antibodies

Antibodies used	BD Pharmingen™ Purified Mouse Anti- BrdU, BD Biosciences, cat# 555627, clone 3D4, lot 7033666<br>Mouse monoclonal antibody Anti-Histone (Merck, Cat# MAB3422, clone H11-4, Chemicon®)
Validation	Purified Mouse Anti- BrdU: this is a monoclonal antibody that has been used for DNA replication/cell proliferation assay for decades. In addition to the QC described in supplier's datasheet, we confirm the antibody specificity by quantifying the marker loci's DNA from immuno-precipitated nascent DNA from BrdU pulse-labeled cells.<br><br>Anti-histone antibody: validated by vendor via immunohistochemistry and western blotting.

Eukaryotic cell lines

Policy information about cell lines and Sex and Gender in Research

Cell line source(s)	Stem cells (H1), hTert-immortalized human foreskin fibroblasts (HFF), and chronic myelogenous leukemia lymphoblasts (K562) were obtained from the 4D Nucleome (4DN) Cell Repository and cultured following the 4DN Consortium's approved culture protocol for each cell line (https://www.4dnucleome.org/cell-lines.html).<br><br>F121-9 from Rudolf Jaenisch lab at Whitehead Institute for Biomedical Research, female<br>HCT116 from ATCC, male<br><br>For scHi-C data generation<br>- The modified WTC-11 (GM25236) hiPS cell line with GFP tagged AAVS1 locus: iPSC from Fibroblast, male, 30YR at sampling
Authentication	No specific authentication of cell lines was perfored. Note that we did perform RNAseq for all lines, and the data confirmed cell type (H1-hESC markers expressed in H1-hESC but not in HFFc6, and vice versa).<br><br>The cell lines in the 4DN Cell Repository were established by the 4DN Consortium in collaboration with WiCell and ATCC for providing quality-controlled cells from the identical batch to minimize cell source and culture condition variations. The cell culture protocols were developed by the 4DN Cell Line Working Group and approved by the 4DN Steering Committee.<br><br>For scHi-C data generation<br>- The modified WTC-11 hiPS cell line with GFP tagged AAVS1 locus (clone 6 and clone 28) was recommended by 4DN Consortium.
Mycoplasma contamination	Negative of mycoplasma from genomic sequencing data.<br><br>For scHi-C data generation<br>- The modified WTC-11 hiPS cell line with GFP tagged AAVS1 locus (clone 6 and clone 28) was tested negative for mycoplasma contamination.

Commonly misidentified lines (See ICLAC register)	None of the cell lines used are on the list of commonly misidentified cell lines.

Plants

Seed stocks	This not relevant to our study.
Novel plant genotypes	This not relevant to our study.
Authentication	This not relevant to our study.

Flow Cytometry

Plots

Confirm that:

- [ ] The axis labels state the marker and fluorochrome used (e.g. CD4-FITC).
- [ ] The axis scales are clearly visible. Include numbers along axes only for bottom left plot of group (a 'group' is an analysis of identical markers).
- [ ] All plots are contour plots with outliers or pseudocolor plots.
- [ ] A numerical value for number of cells or percentage (with statistics) is provided.

Methodology

Sample preparation	Formaldehyde-crosslinked WTC-11 cells were lysed and permeabilized with 0.5% SDS. The lysates were then incubated overnight at 37 °C with 300 U of MboI, followed by proximity ligation using T4 DNA ligase at room temperature with gentle rotation for 4 hours. After staining with Hoechst, the nuclei were subjected to fluorescence-activated cell sorting (FACS).
Instrument	SH800 cell sorter (Sony Biotechnology)
Software	SH800 software (Sony Biotechnology)
Cell population abundance	Due to aggregation of nuclei caused by the Hi-C preprocessing steps, fewer than 10% of WTC-11 nuclei remained as single, isolated nuclei that could be sorted for downstream library preparation.
Gating strategy	Single nuclei were identified and gated based on FSC-A versus FSC-W parameters to distinguish singlets from aggregates. The Hoechst fluorescence channel was then used to differentiate 2N nuclei from those in replication or mitotic phases.

- [ ] Tick this box to confirm that a figure exemplifying the gating strategy is provided in the Supplementary Information.
